# Quality of Essential Medicines from Different Sources in Enugu and Anambra, Nigeria

**DOI:** 10.4269/ajtmh.23-0837

**Published:** 2024-05-14

**Authors:** Julia Gabel, Micha Lächele, Katharina Sander, Gesa Gnegel, Nkiru Sunny-Abarikwu, Rita Ezinwanne Ohazulike, Juliet Ngene, Jane Frances Chioke, Christine Häfele-Abah, Lutz Heide

**Affiliations:** ^1^ Pharmaceutical Institute, Eberhard Karls University Tübingen, Tübingen, Germany;; ^2^ Faith-Based Central Medical Foundation (FBCMF), Enugu, Nigeria;; ^3^ German Institute for Medical Mission (Difaem), Tübingen, Germany

## Abstract

This study investigated the quality of 13 essential medicines in the states of Enugu and Anambra, Nigeria. A total of 260 samples were purchased from licensed pharmaceutical manufacturers and wholesalers and from vendors in pharmaceutical markets with unclear licensing status. Samples were analyzed for identity, content, and dissolution according to the United States Pharmacopeia (USP) 42 monographs. Forty-five samples of this study could be examined for authenticity with the Mobile Authentication Service scheme of the Nigerian National Agency for Food and Drug Administration and Control. Out of all samples, 25.4% did not comply with the USP 42 specifications. Strikingly, 21 out of 22 dexamethasone tablet samples (95%) were out of specification (OOS). Nine out of 19 glibenclamide samples (47%) failed dissolution testing, and 7 out of 17 cotrimoxazole samples (41%) failed assay testing. Medicines against noncommunicable diseases showed a slightly higher percentage of OOS samples than anti-infectives (21.2% versus 17.6%). The rates of OOS samples were similar in medicines stated to be produced in Nigeria, India, and China but were very different between individual manufacturers from each of these countries of origin. Therefore, prequalification of products, manufacturers, and suppliers are very important for quality assurance in medicine procurement. Unexpectedly, the total proportions of OOS samples were similar from licensed vendors (25.2%) and from markets (25.5%). Four samples (1.5%), all collected in markets, were clearly falsified and did not contain the declared active pharmaceutical ingredients. The proportion of falsified medicines was found to be lower than frequently reported in the media for Nigeria.

## INTRODUCTION

The United Nations demand in their Sustainable Development Goals “access to safe, effective, quality and affordable essential medicines” for all.^1^ However, the achievement of this goal is compromised by the frequent occurrence of substandard and falsified (SF) medicines. As defined by the WHO,^2^ falsified medicines are “medical products that deliberately/fraudulently misrepresent their identity, composition or source.” Substandard medicines are “authorized medical products that fail to meet either their quality standards or their specifications, or both.” Substandard and falsified medicines can lead to increased mortality and morbidity but also to economic loss and increased poverty.^2^ Furthermore, underdosed anti-infectives can contribute to the emergence of antimicrobial resistance.^3^

Especially low- and middle-income countries (LMICs) suffer from SF medicines. A systematic review by the WHO from 2017^2^ estimated a prevalence of 10.5% SF medicines in LMICs. A more recent meta-analysis by Ozawa et al.^4^ estimated an overall prevalence of 12.4% of SF medicines in all LMICs and 18.9% in African countries. Another review calculated a prevalence of even 25%.^5^ However, all these reviews state that the reported prevalence rates are very heterogeneous between individual studies and that more consistent data on the quality of medicines are urgently required. Such data could support policymakers on the best use of resources to tackle the problem of SF medicines and to implement the three-pronged strategy of prevention, detection, and response suggested by the WHO.^6^

Heterogeneous findings on the prevalence of SF medicines have also been reported from Nigeria. Table 1 summarizes the results of the 11 most important studies published since 2001. These studies investigated different types of medicines and used high-performance liquid chromatography (HPLC) for analysis. The reported percentages of samples not containing the declared active pharmaceutical ingredient (API) ranged from 0% to 4%. Importantly, the reported percentages of substandard samples, showing incorrect amounts or insufficient dissolution of the API, ranged from 1.3% to 74%, with a median value of 29% that markedly exceeds the overall estimate for LMICs in the WHO review mentioned above.^2^

**Table 1:** Previous studies on SF medicines in Nigeria

Study	Type of Investigated Medicines	Parameters Investigated (Specifications Used)	No. of Samples Investigated	No. (%) of Falsified Samples Not Containing the Stated API(s)	No. (%) of Substandard Samples	Comment
Taylor et al. (2001)^71^	Anti-infectives	Assay (BP)	581	6 (1.0)	273 (47)	Somewhat unusually, most of the substandard samples were reported to contain an excessive amount of API.
Onwujekwe et al. (2009)^72^	Antimalarials	Dissolution profile; API amount* (USP)	225	9 (4.0)	51 (23)	Abstract and text state “37% did not meet USP specification”; however, this is a calculation error.
WHO (2011)^43^	Antimalarials	Assay; dissolution (USP, Ph. Int.)	61^†^	2 (3.3)^†^	29 (48)^†^	15 samples (25%) showed deviations classified as “extreme” based on the criteria also used in the present study (see “Definitions of medicine quality”). Related substances and uniformity of mass of dosage units were investigated as well.
Ebenezer (2015)^73^	Metformin	Assay (BP)	179	0 (0)	7 (3.9)	The study compared the originator product Glucophage® (Merck KGaA, Darmstadt, Germany) with generic medicines. All originator samples were in specifications.
WHO (2016)^74^	Medicines for maternal health	Assay (USP, BP, Ph. Int.)	17^†^	0 (0)^†^	5 (29)^†^	
Kaur et al. (2016)^75^	Artemisinin-based combinations	Assay (limits 85–115%)	3,024	(1.2)	(6.6)	Only percentages, not numbers of SF medicines, are given. Assay tolerance limits are wider than those of USP and may lead to a 3-fold-lower prevalence estimate compared with that of studies using USP specifications.^39^
Anyakora et al. (2018)^76^	Medicines for maternal health	Assay (USP)	Oxytocin, 159; misoprostol,166	Oxytocin, 1 (0.6); misoprostol, 0 (0)	Oxytocin, 117 (74); misoprostol, 56 (34)	Also investigated magnesium sulfate and calcium gluconate injections, reporting 6.8% and 2.4% of substandard samples, respectively.
Lawal et al. (2019)^77^	Oral antibiotics	Assay (USP, BP)	112	3 (2.7)	40 (36)	
Redfern et al. (2019)^78^	Amlodipine, lisinopril	Assay (USP)	361	0 (0)	101 (28)	
Ndichu et al. (2019)^79^	Nifedipine	Assay (Ph. Int.)	102	0 (0)	30 (29)	
NAFDAC (2019)^20^	Antimalarials	GPHF-Minilab; assay (USP?)	907	0 (0)	12 (1.3)	All samples were analyzed with GPHF-Minilab. Samples failing Minilab analysis, as well as a certain percentage of samples passing Minilab analysis, were analyzed by HPLC, probably according to USP.

API = active pharmaceutical ingredient; BP = British Pharmacopoeia; HPLC = high-performance liquid chromatography; Ph. Int. = International Pharmacopoeia; SF = substandard and falsified; USP = United States Pharmacopeia.

* Onwujekwe et al. (2009)^72^ calculated the API amount from the dissolution testing experiment.

^†^ Several countries were investigated in these studies, but the numbers shown here are for Nigeria only.

Several other studies^7^^–^^9^ investigated medicine quality in Nigeria using the Global Pharma Health Fund (GPHF)-Minilab, which is based on thin-layer chromatography (TLC).^10^ However, as GPHF-Minilab analysis has a lower sensitivity than HPLC analysis for the detection of substandard medicines,^11^ their results cannot be compared with those of the above-mentioned studies. A few further medicine quality studies included only small sample numbers from Nigeria.^12^^–^^19^

The Nigerian national medicine regulatory agency (National Agency for Food and Drug Administration and Control [NAFDAC]) has conducted medicine quality surveys in collaboration with the United States Pharmacopeia (USP), but the methods and results of these studies have not been published in full scientific detail. The study by NAFDAC included in Table 1 was summarized in a NAFDAC newsletter in 2019.^20^ In that survey, analysis of all samples was carried out with the GPHF-Minilab. Samples failing Minilab analysis, as well as a certain percentage of samples passing Minilab analysis, were subjected to confirmatory assay testing using HPLC.

The above-mentioned studies clearly demonstrate that in Nigeria, as in other LMICs, quality assurance in drug procurement is extremely important for achieving “access to quality medicines for all” as demanded in the Sustainable Development Goals of the United Nations. In Nigeria, as in most other African countries, faith-based organizations provide an important part of the health services to the population, including pharmaceutical services.^21^^–^^23^ Many faith-based drug supply organizations are members of the Ecumenical Pharmaceutical Network (EPN).^21^ The EPN describes itself as an “independent, non-profit, Christian organization committed to provide quality-assured pharmaceutical services”.^24^ In Nigeria, one of the EPN member organizations is the Faith-Based Central Medical Foundation (FBCMF), based in the state of Enugu. The FBCMF procures medicines within Nigeria and supplies them primarily to faith-based health facilities in Enugu and neighboring states. The EPN gives great importance to pharmaceutical quality assurance, and the FBCMF has been an active member of the “Difaem-EPN Minilab Network” since 2017, employing the GPHF-Minilab^10^ for local medicine quality screening.^9^^,^^25^^,^^26^

The present study was undertaken to assist the FBCMF and other stakeholders in Nigeria in the further improvement of their quality assurance in drug procurement. As explained in Materials and Methods, 13 essential medicines important in the FBCMF’s medicine supply operation were chosen, comprising both medicines against infectious diseases and medicines against noncommunicable diseases (NCDs). Because the sampling for the present study was conducted during the COVID-19 pandemic, two medicines with alleged or real relevance for the treatment of COVID-19 were included, i.e., chloroquine and dexamethasone, respectively. All these medicines were purchased in Nigeria from a total of 62 different commercial sources, including licensed manufacturers and wholesalers as well as pharmaceutical “markets” of unclear licensing status. The quality of the medicines was investigated locally by FBCMF staff using the GPHF-Minilab and at Tübingen University, Germany, according to the USP for the content and dissolution of the APIs. The registration numbers of the medicines were compared with Nigeria’s Registered Drug Product Database (the “NAFDAC Greenbook”).^27^ Medicines carrying a personal identification number (PIN) code of NAFDAC’s Mobile Authentication Service (MAS) scheme^28^ were tested for the authenticity information provided by this scheme using short messaging service (SMS) messaging in Nigeria.

The quality of the investigated medicines was found to be very different between different types of medicines and between different manufacturers, and these results may be useful for the further improvement of pharmaceutical quality assurance by the FBCMF and other stakeholders in Nigeria and elsewhere.

## MATERIALS AND METHODS

### Study design and ethical approval.

The study design is based on the guidelines on the conduct of surveys of the quality of medicines published by the WHO in 2016^29^ and the Medicine Quality Assessment Reporting Guidelines.^30^ The study protocol was submitted to the Enugu State Commissioner of Health, and permission for this study was granted on January 30, 2021.

### Included medicines.

Thirteen medicines were included in this study (Table 2). The medicines were selected based on their compliance with several or all of the following criteria: 1) inclusion in the Nigeria Essential Medicines List 2020^31^; 2) availability of a monograph in the USP 42 for compendial analysis; 3) availability of a monograph for their analysis with the GPHF-Minilab,^32^ including the possibility for their detection by TLC using ultraviolet (UV) light (254 nm); 4) economic importance in the medicine procurement and distribution by the FBCMF; 5) inclusion in a previous study of our group in Cameroon and the Democratic Republic of the Congo (DRC),^11^ to allow a comparison of results; and 6) alleged or true relevance for the treatment of COVID-19.^33^^,^^34^ Based on advice by the FBCMF, the most common dosage forms and strengths were selected to be preferably sampled. If the medicine was not available in the form of tablets at a sampling site, capsules could be sampled, and vice versa. If the preferred strength was not available, a different strength could be sampled but only adult dosages. Injectables and oral dosage forms could not be substituted for each other.

**Table 2:** Overview of medicines included in this study

API	Preferred Dosage Form	Preferred Strength (mg)	No. of Samples Collected from Vendors in Pharmaceutical Markets with Unclear Licensing Status	No. of Samples Collected from Licensed Pharmaceutical Manufacturers and Wholesalers
Atenolol^†^	Tablet	50	10	4
Ceftriaxone sodium	Powder for injection	1000	11	12
Cefuroxime axetil	Tablet	250	13	11
Chloroquine phosphate (or sulfate)	Tablet	250 (or 200)	10	6
Ciprofloxacin hydrochloride^†^	Tablet	500	14	12
Dexamethasone	Tablet	4*	14	8
Fluconazole	Capsule^‡^	150	12	10
Furosemide^†^	Tablet	40	12	2
Glibenclamide^†^	Tablet	5	9	10
Hydrochlorothiazide^†^	Tablet	25	11	5
Metformin hydrochloride^†^	Tablet	500	12	10
Metronidazole^†^	Tablet	200^§^	11	14
Cotrimoxazole^†^	Tablet	480	10	7
Total			149	111
Grand total			260	

API = active pharmaceutical ingredient.

* None of the dexamethasone samples could be obtained in the form of 4 mg tablets. Rather, 18 samples were obtained as 0.5 mg tablets, and four samples were obtained as 1 mg tablets.

^†^ Medicine types also included in a previous study of our group in Cameroon and the DRC.^11^

^‡^ Seven fluconazole samples could be obtained as capsules, and the other 15 could be obtained as tablets.

^§^ Twenty-two metronidazole samples could be obtained as 200 mg tablets, and the other three could be obtained as 400 mg tablets.

### Sample size calculation.

In a previous study in Cameroon and the DRC,^11^ 12.3% of the medicine samples from health facilities in the formal sector had been found to be noncompliant with pharmacopeial specifications, in contrast to 28.2% of the samples from informal vendors. Using these proportions, the minimum sample size required to observe a significant difference between these groups with 95% confidence and a power of 80% was calculated as 97 samples per group, applying the formula^35^

$$n={\left(Z_{\frac{\alpha }{2}}+Z_{\beta }\right)}^{2}\times (p_{1}\left(1-p_{1}\right)+p_{2}\left(1-p_{2}\right))/{\left(p_{1}-p_{2}\right)}^{2}$$

where *n* is the minimum sample size required, *Z*_α/2_ is the critical value of the normal distribution at α/2, i.e., 1.96 for a 95% confidence level (α = 0.05), $Z_{\beta }$ is the critical value of the normal distribution at β, i.e., 0.84 for a power of 80% (β = 0.2), and *p*_1_ and *p*_2_ are the expected sample proportions of the two groups, i.e., 12.3% and 28.2%, respectively.

It was therefore decided to attempt collection of 10 samples of each of the 13 medicines from licensed manufacturers and wholesalers and another 10 samples each from pharmaceutical “markets” with unclear licensing status, resulting in a theoretical number of 130 samples per group.

### Number of units purchased for each sample.

For each sample, if possible, 100 tablets/capsules, or 20 vials in the case of ceftriaxone injections, were purchased. If the medicine was sold only in packages larger than 100 tablets/capsules or 20 vials, the entire package was purchased to obtain the original packaging, provided the expense for a single sample did not exceed 20,000₦ (approximately $50). If only a smaller amount than 100 tablets/capsules or 20 vials was available, this smaller amount was collected, but not fewer than 30 tablets/capsules or five vials per sample.

### Sampling sites and sample collection.

Medicines were collected from two types of sources: 1) licensed pharmaceutical manufacturers and wholesalers (hereafter referred to as licensed vendors) and 2) vendors in pharmaceutical markets of Onitsha and Enugu with unclear licensing status (hereafter referred to as markets).

As part of its medicine procurement and distribution operation, the FBCMF keeps a list of licensed vendors. For the present study, the 74 vendors on the list at that time were contacted by the FBCMF staff using WhatsApp for the procurement of the 13 medicine types listed in Table 2, without mentioning the intended medicine quality testing. Medicines were subsequently bought from those suppliers who could most readily deliver the requested items. Most licensed vendors were able to offer only one or a few of the 13 medicine types requested.

The market in Onitsha (Anambra State) is well known as one of the largest cluster of market vendors of medicines in Nigeria.^36^^–^^38^ These vendors operate in professional-looking premises, but their status of licensing is unclear. A similar but much smaller market is located in Enugu town (Enugu State). Even private hospitals and pharmacies frequently buy medicines from these markets.^36^ Occasionally, the FBCMF also needs to purchase medicines from these markets, especially when a certain product is unavailable from the licensed vendors. For the present study, four FBCMF staff members visited Onitsha and Enugu pharmaceutical markets to purchase the study medications, again without mentioning the intended medicine quality testing. No questions were asked by the market vendors about the reasons for the purchase. The FBCMF staff members selected the market vendors for this study based on convenience, first visiting the largest market vendors in Onitsha market and subsequently neighboring ones. Sample collection was carried out first in the Onitsha market and subsequently in the Enugu pharmaceutical market.

Especially the larger market vendors were able to offer several of the 13 medicine types requested and often more than one brand of each type of medicine. All available brands were purchased until the desired number of samples was reached. Because several FBCMF staff members were carrying out the purchases in parallel, the targeted number of 10 samples per medicine type was exceeded in several cases (Table 2).

A first round of sample collection was conducted from July to August 2021. Because the targeted number of samples had not yet been reached from the licensed vendors, a second round of sample collection was carried out from February to June 2022.

When samples were procured from the licensed suppliers, payment of the samples was conducted in accordance with the standard procedures of the FBCMF. For the Onitsha and Enugu pharmaceutical markets, the collected medicines were paid for in cash by the investigators.

### Sample handling and shipment.

Samples were collected in their original containers whenever possible. Otherwise, they were collected in light- and air-tight screw-cap plastic containers carried by the investigators. Containers that were not full were filled up with clean cotton wool to minimize mechanical damage to the tablets/capsules during transport. Upon collection, each sample was labeled immediately with a unique code number using preprinted adhesive sample labels. As soon as possible after sample collection, each sample was photographed from all sides.

All obtained samples were transported to the air-conditioned medicine storage rooms of the FBCMF without delay, where they were stored at 20°C. For each sample, 25 tablets or capsules (or five vials in the case of ceftriaxone injections) were kept by the FBCMF for on-site GPHF-Minilab analysis. The remaining samples were shipped by commercial courier service to Tübingen University in November 2021 for samples from the first round of sample collection and in June 2022 for samples from the second round. Upon their arrival at Tübingen University, they were stored in an air-conditioned room at 21°C until analysis.

### Chemical analysis.

Compendial analysis for identity and quantity (i.e., assay) of the APIs was carried out at the Pharmaceutical Institute of Tübingen University according to the USP 42 monographs, in accordance with the recommendation by Hauk et al.^39^ High-performance liquid chromatography was performed with an Agilent 1260 Infinity II system and an Agilent 1100 system (Agilent Technologies, Santa Clara, CA). Columns for HPLC analysis were obtained from A. Maisch HPLC GmbH (Ammerbuch-Entringen, Germany), and certified pharmaceutical secondary standards were obtained from Sigma-Aldrich (St. Louis, MO). For furosemide tablets, the HPLC column and solvent system described in the British Pharmacopoeia 2022 were used.

For dissolution testing, the analytical procedures described in the respective monographs of USP 42 were followed, using a PTWS 610 dissolution tester (Pharma Test Apparatebau AG, Heinburg, Germany) and an Agilent 708-DS dissolution apparatus (Agilent Technologies). Quantification of the dissolved APIs was carried out using the HPLC systems described above or by UV spectroscopy (for cefuroxime axetil, chloroquine, and furosemide) using a Perkin Elmer Lambda 25 UV/Vis spectrometer. The USP published in 2021 its intent to revise the method for dissolution testing of dexamethasone tablets,^40^ and this revised method was followed. One metformin sample represented sustained-release tablets, and the respective USP dissolution specifications (Supplemental Table S1) were observed for that sample. For stage *S*_1_ of dissolution testing, the number of units tested and the lower limits for average and minimum dissolution rates (Supplemental Table S1) followed the method for small-scale dissolution screening published by Rahman et al.^41^ As described by Rahman et al.,^41^ for samples failing stage *S*_1_, any subsequent stage *S*_2_ dissolution testing followed USP 42 specifications, both for the total number of units tested and for the lower limits for average and minimum dissolution. No stage *S*_3_ testing was carried out.

Dissolution testing was not carried out for the 23 ceftriaxone samples, as these represented powders for injection, and not for the seven fluconazole capsule samples because no dissolution testing method for fluconazole capsules is specified in the USP 42. Furthermore, for one ciprofloxacin sample, not enough tablets were available for dissolution testing.

Content uniformity was not assessed in the present study because of the limited resources available.

### Definitions of medicine quality.

In this study, the current definitions of SF medicines by the WHO were used,^42^ together with additional criteria suggested by Hauk et al.^39^ and by Ozawa et al.^4^ As proposed in the WHO QAMSA study^43^ and applied in a previous study by our group in Cameroon and the DRC,^11^ samples deviating from USP specifications were further divided into those showing moderate deviations from the pharmacopeial limits and those showing extreme deviations. Extreme deviations were defined as deviations of more than 20% from the stated amount of API in assay analysis and/or average dissolution rates of the API of the tested units falling more than 25% below the pharmacopeial threshold (i.e., falling below the pharmacopeial *Q* value minus 25%).

### Statistical calculations.

Calculations and statistical analyses were conducted using Excel 2019 (included in Microsoft 365; Microsoft Corp., Redmond, WA). For calculation of significant differences between proportions, MedCalc® (MedCalc Software Ltd., Ostend, Belgium) was used.^44^

### Information of national authorities and stakeholders.

The Enugu State Commissioner of Health (Nigeria), the NAFDAC, and the WHO Global Surveillance and Monitoring System for SF medical products were informed about the results of the study.

## RESULTS

### Overview of collected medicines.

Two hundred fifty-seven medicine samples were purchased from 62 commercial sources. However, visual inspection showed that three purchased samples included blisters of two different batches, instead of representing a uniform sample. These different batches were subsequently treated as separate samples and analyzed for their quality individually. Therefore, the total number of investigated samples was 260. The number of samples collected from licensed vendors and from markets for every API is depicted in Table 2. Nearly all samples could be obtained in the preferred dosage form and strength; exceptions are noted in the footnotes of Table 2.

Nearly all collected samples were generic products, either sold under their international nonproprietary names (“unbranded generics”) or sold under a brand name decided by the marketing authorization holder (“branded generics”). Only three out of the 260 samples (1%) represented originator products; all three were found to comply with pharmacopeial specifications. No expired products were encountered during sample collection.

**Figure 1.**
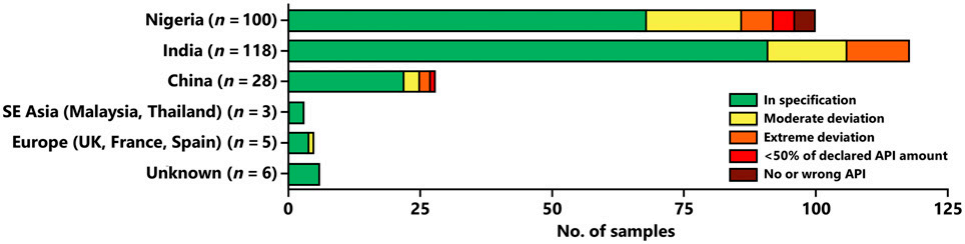
Number of samples stated to be manufactured in different countries and results of their compendial quality testing. See the legend for Figure 5 for definitions of quality categories. The four discovered falsified medicines (containing no active pharmaceutical ingredient [API] or a wrong API) were stated to be manufactured in Nigeria, but this statement might be incorrect.

The 260 samples collected in this study were manufactured by 89 different manufacturers located in eight different countries. As illustrated in Figure 1, 45.4% of the samples were stated to be manufactured in India, 38.5% in Nigeria, and 10.8% in China. In contrast, only 1.9% of the samples were stated to be manufactured in Europe (United Kingdom, France, and Spain) and 1.2% in Southeast Asia (Malaysia and Thailand). For nine samples, a marketing authorization holder but no manufacturer was stated; however, for three of these, representing the same brand, the country of manufacture was stated (India). All nine were found to comply with pharmacopeial specifications.

Detailed information on all samples, with their stated manufacturers and countries of manufacture, is given in Supplemental Table S2.

### Falsified medicines.

**Figure 2.**
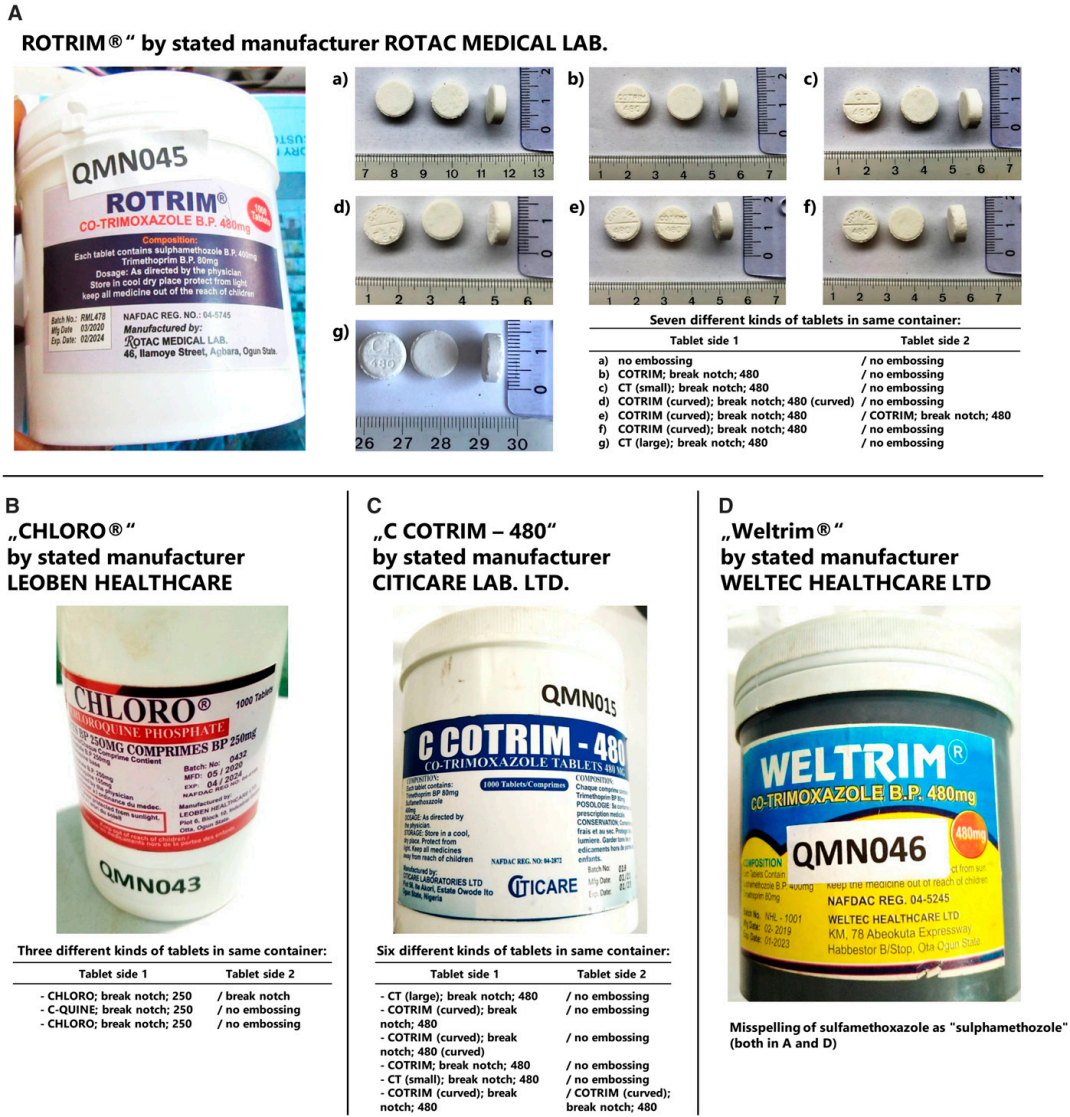
Four falsified medicines discovered in this study. Products A, B, and C contained different kinds of tablets, with different embossings and thicknesses, in the same container. None of these contained any active pharmaceutical ingredient. Product D contained paracetamol (27 mg per tablet) instead of the labeled ingredients sulfamethoxazole and trimethoprim.

Within the 260 investigated samples, four (1.5%) did not contain the stated API(s) (Figure 2A to D). All four samples had been obtained in markets, not from licensed vendors. One of them was labeled as containing chloroquine phosphate 250 mg, and the other three were labeled as containing sulfamethoxazole/trimethoprim 400/80 mg. All four represented remarkably crude falsifications: the samples depicted in Figure 2A, B, and C contained several different kinds of tablets with different embossings and different thicknesses within the same bulk container. On the labels of the samples depicted in Figure 2A and D, the API sulfamethoxazole was misspelled as “sulphamethozole.” Although all four product labels showed a NAFDAC registration number, three of these could not be found in Nigeria’s Registered Drug Product Database,^27^ and the fourth one belonged to a completely different product in the database. The names of the four stated manufacturers could not be found either in an internet search or in Nigeria’s Registered Drug Product Database.^27^ Apparently, the stated manufacturers do not exist. Notably, the name of one of these manufacturers, Citicare Laboratories Ltd. (Figure 2C), has been detected previously on falsified medicines in Nigeria.^20^

High-performance liquid chromatography analysis showed that three of these products did not contain any detectable amount of API. In contrast, the product labeled “Weltrim” (Figure 2D) was found not to contain sulfamethoxazole and trimethoprim but instead paracetamol (27 mg per tablet), identified by HPLC, TLC, and UV analysis in comparison with an authentic paracetamol reference standard. Falsified medicines containing low amounts of paracetamol have also been found in previous studies.^11^^,^^33^

Two further samples were found to carry a remarkably misspelled logo, depicted in Supplemental Figure S1 and stating “WHO GMP CERTIFIED QALITY [sic] PRODUCT”. Although the WHO has published guidelines for the issuance of good manufacturing practice (GMP) certifications by national authorities, the WHO itself does not issue such certificates. Both samples represented the same batch and brand of “Eden Fluconazole 150 mg Capsules” (stated manufacturer, Impulse Pharma Pvt. Ltd., India). The stated NAFDAC registration number (C4-0072) could not be verified in Nigeria’s Registered Drug Product Database,^27^ but the marketing authorization holder, Eden U.K Pharmaceutical Ltd., Nigeria, was represented in that database with several other products. Both samples complied with USP specifications for the content of the API (dissolution was not tested for fluconazole capsules; see Materials and Methods). It was therefore decided to consider these two samples not as falsified but as “in specification.”

### Analysis of the quantity of the APIs.

**Figure 3.**
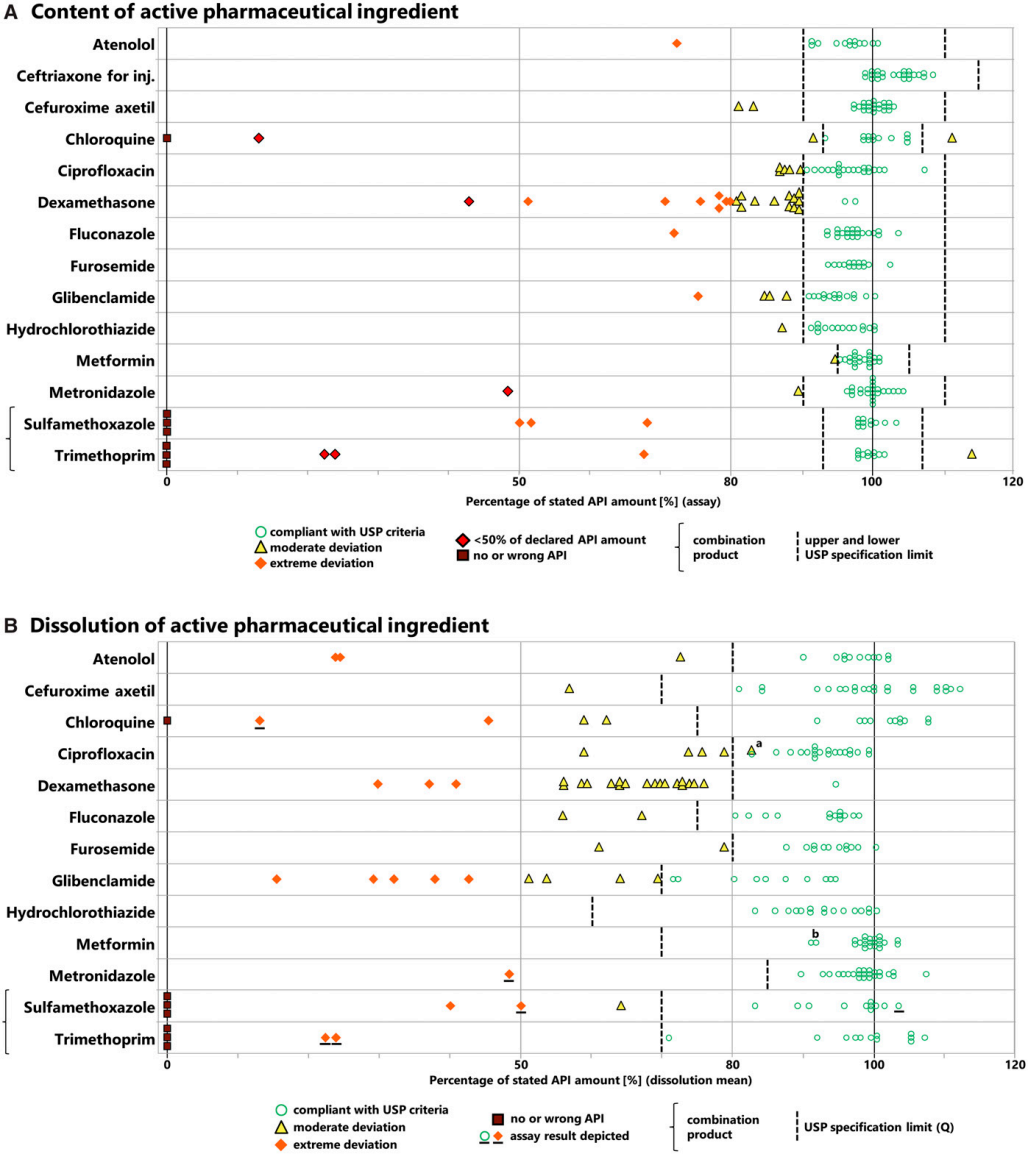
Content and dissolution of the active pharmaceutical ingredient (API) determined for each sample, expressed as a percentage of the stated API amount. (**A**) Content of the API. (**B**) Dissolution of the API. The specification limits of the United States Pharmacopeia (USP) (*Q* values in case of dissolution) are depicted. ^**a**^For this ciprofloxacin sample, individual tablets showed a dissolution of less than *Q* minus 15%. Thereby, this sample deviates from USP specifications, even though its mean dissolution is above the pharmacopeial *Q* value of 80%. ^**b**^This metformin sample was formulated as an extended-release tablet. Therefore, dissolution testing was carried out and evaluated according to the USP monograph for metformin extended-release tablets (see Supplemental Table S1). Dissolution testing was not carried out for four samples that already showed in assay analysis contents of chloroquine, metronidazole, or trimethoprim lower than *Q* minus 25% (see text). Their API contents are depicted in Figure 3B with underlined symbols.

All 260 collected samples were subjected to assay analysis (i.e., quantification of API content). Figure 3A shows the result for each sample as a percentage of the API amount stated on the label. The USP specifies compliance limits for each API, as depicted in Figure 3A and summarized in Supplemental Table S1. Within the investigated medicines, these limits ranged from 90% to 115% of the declared amount for ceftriaxone injections up to 95% to 105% for metformin tablets. Among all 260 samples, 212 (81.5%) showed an API amount within the USP specifications. Twenty-seven samples (10.4%) showed a moderate deviation (i.e., deviations not exceeding ±20% of the stated amount). Twenty-one samples (8.1%) showed an extreme deviation, i.e., a deviation of more than 20% of the stated amount. The latter 21 samples comprised 12 samples containing between 50% and 79.9% of the stated API amount, 5 samples containing less than 50% of the stated API amount, and 4 samples not containing the stated APIs at all.

Samples not containing the stated APIs at all were considered falsified (see above). In accordance with the suggestion by Hauk et al.^39^ and Ozawa et al.,^4^ samples containing less than 50% of the stated API amount, without evidence that their low content may have been due to API degradation, were considered “probably falsified” because it does not appear likely that such deviations can occur without fraudulent intent.

Photos of the five probably falsified samples are depicted in Supplemental Figure S2A to E. The product shown in that figure (stated name, “SA’A QUINE,” chloroquine phosphate tablets) was found to contain only 13.1% of the stated API amount. Notably, for this product, the batch numbers and expiry dates on the secondary packaging were different from those on the blisters, and even different ones appeared on different blisters.

The two samples shown in Supplemental Figure S2B and C (stated names, “Poletrim” and “Zimatrim”) were labeled to contain sulfamethoxazole/trimethoprim 400/80 mg but contained only 22.4% and 23.9% of the declared amount of trimethoprim, respectively. The first of these two samples furthermore contained only 50.1% of the declared amount of sulfamethoxazole, whereas the latter one contained the correct amount of that API.

A fourth product was labeled “Zunagyl” (Supplemental Figure S2D) and contained only 48.4% of the declared amount of metronidazole. The tablets of this product exhibited an unpleasant odor. All four above-mentioned products were stated to be produced by Nigerian manufacturers listed in NAFDAC’s List of Inspected Local Pharmaceutical Manufacturing Facilities,^45^ but only two of these were still listed in Nigeria’s Registered Drug Product Database^27^ in September 2023.

A fifth product, labeled “Destrax” (Supplemental Figure S2E), was a dexamethasone tablet product stated to be manufactured in China. It contained only 42.9% of the stated amount of the API.

Among the 260 samples, there were 22 cases in which two samples of the same brand and batch had been collected, and in an additional three cases, even three samples of the same batch had been collected. As expected, in most of these cases (i.e., in 22 out of 25 cases), the different samples of the same batch showed very similar assay results: the assay values of the individual samples deviated from the mean assay value for the respective batch by ±0.9% on average. However, there were three notable exceptions. First, three samples of chloroquine phosphate tablets with the stated name “Quimal” (stated manufacturer, Dana Pharmaceuticals Limited, Nigeria; stated batch number, QT145) showed assay results of 98.9%, 98.8%, and 111.4% of the stated API amount. The excessive API content of the latter sample was also confirmed in the dissolution testing. This amount exceeds the pharmacopeial limit of 107% (Supplemental Table S1), and the unequal contents of samples of the same batch indicates serious violations of GMP.

Similarly, two samples of glibenclamide tablets with the stated name “Tionil” (stated manufacturer, Merit Organics Ltd., India; stated batch number, T32002) showed very different assay results, i.e., 94.7% and 75.4% of the stated API amount. Both samples also failed in dissolution testing, the latter sample showing a dissolution of only 42.7% of the stated API amount.

Finally, two samples of chloroquine phosphate tablets named “Albequine” (stated manufacturer, Alben Healthcare Ind. Ltd., Nigeria; stated batch number, 017) showed assay results of 99.3% and 91.7% of the declared amount, respectively; the latter value is below the pharmacopeial limit of 93.0%. This latter sample was found to contain, within the same blister packs, both white tablets and tablets with brown spots. A subsequent separate assay analysis of these two types of tablets resulted in 100.6% and 81.9% of the stated API amount, respectively. Both investigated samples of this batch failed in dissolution testing. It cannot be excluded that the observed brown spots are the result of microbial contamination.^46^^,^^47^

### Analysis of the dissolution of the APIs.

Of the 260 collected samples, 229 were eligible for dissolution testing as described in Materials and Methods. Two hundred twenty-one of those 229 samples were subjected to dissolution testing, and Figure 3B shows the result for each sample. The USP specifies a compliance limit (*Q* value) for each API, as depicted in Figure 3B and summarized in Supplemental Table S1. Within the investigated medicines, these limits ranged from a dissolution of ≥60% of the declared API amount for hydrochlorothiazide tablets to ≥85% for, e.g., metronidazole tablets.

Among the 221 investigated samples, 173 (78.3%) showed an API dissolution complying with the USP specifications. Thirty-six samples (16.3%) showed a moderate deviation (i.e., an average dissolution between *Q* and *Q* minus 25%). Twelve samples (5.4%) showed an extreme deviation (i.e., an amount of the dissolved API more than 25% lower than the pharmacopeial *Q* value).

Dissolution testing was not carried out for the four falsified medicines that did not contain the declared APIs. Furthermore, it was decided not to carry out dissolution testing for one chloroquine, one metronidazole, and two sulfamethoxazole/trimethoprim samples, because for these samples, the above-described assay testing had already shown an API content that was more than 25% lower than the pharmacopeial *Q* value. Their API contents are depicted in Figure 3B with underlined symbols. Inclusion of these samples into the above calculation increases the number of samples not complying with the USP dissolution specifications to a total of 56 out of 229 samples (24.5%).

As already observed for the assay values, different samples of the same batch showed similar dissolution results. However, there was one notable exception (beyond the cases with different assay results in the same batch, described above): two samples of glibenclamide tablets with the stated name “Glanil” (stated manufacturer, Nigerian-German Chemicals Plc; stated batch number, FPD070421) showed very different dissolution results, i.e., 64.1% and 32.1% of the stated amount of the API, respectively. Both values are below the pharmacopeial *Q* value of 70%. Notably, on the blisters of the second sample, the name of the API was misspelled as “gilbenclamide,” whereas the spelling was correct on the blisters of the first sample, carrying the same batch number. Three further batches of this “Glanil” brand were investigated in this study, all of them failing dissolution testing, with only 29.2%, 37.9%, and 51.2% of the API being dissolved. One of these samples (stated batch number, FPD070321) also showed the misspelling “gilbenclamide” on the blister. These observations indicate severe shortcomings in the manufacturing of this product.

Samples representing the same brand (albeit different batches) showed mostly consistent results regarding compliance or noncompliance with dissolution testing. However, six brands were found where some batches passed dissolution testing and others did not. The most prominent examples were five samples of “Eden Atenolol” (stated manufacturer, Impulse Pharma Pvt. Ltd., India), showing dissolution rates of 96.3%, 95.9%, 94.7%, 24.5%, and 23.8%, respectively; the last two values represent extreme deviations from the pharmacopeial *Q* value of 80%. Furthermore, two samples of “Biocipro” (stated manufacturer, McCoy Pharma Pvt. Ltd., India) showed dissolution rates of 91.8% and 59.0%, respectively; the latter value falls below the pharmacopeial *Q* value for ciprofloxacin of 80%.

### Combined results of compendial analysis.

**Figure 4.**
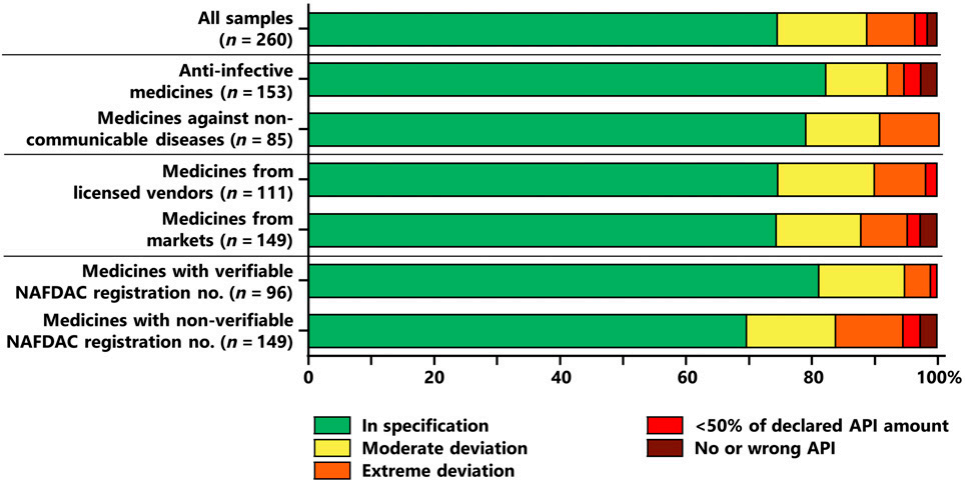
Results of compendial analysis for different therapeutic groups, for different sources of medicines, and for medicines with verifiable and nonverifiable National Agency for Food and Drug Administration and Control (NAFDAC) registration numbers (see text). Dexamethasone can be used in the treatment of both COVID-19 and noncommunicable diseases and was therefore excluded from the comparison of anti-infective medicines with medicines against noncommunicable diseases. See the legend for Figure 5 for definitions of quality categories.

If the results of assay and dissolution testing are combined, 194 of all 260 analyzed samples (74.6%) complied with USP specifications. Thirty-seven samples (14.2%) showed moderate deviations, and 29 samples (11.2%) showed extreme deviations (Figure 4). The latter group comprised five samples (1.9%) that were considered “probably falsified” because their API content was below 50% of the stated amount and four samples (1.5%) that were considered “falsified” because they did not contain any of the stated APIs at all.

Out of the 260 samples, 48 (18.5%) were already found to be out of specification (OOS) by assay testing. However, 18 samples (6.9%) were found to be OOS only by subsequent dissolution testing. This shows that an omission of dissolution testing in medicine quality studies leads to a quite substantial underestimation of the prevalence of substandard medicines.

As shown in Figure 4, medicines against NCDs showed a slightly higher percentage of OOS samples than anti-infective medicines (21.2% versus 17.6%, respectively). Dexamethasone had been excluded from this comparison because it can be used in the treatment of both COVID-19 and NCDs. Contrary to our expectations, medicines obtained from licensed vendors showed an overall proportion of OOS samples similar to that of medicines obtained from markets (25.2% and 25.5%, respectively). However, the four falsified medicines that did not contain the declared APIs were all obtained from markets.

### Results of compendial analysis for the different APIs.

**Figure 5.**
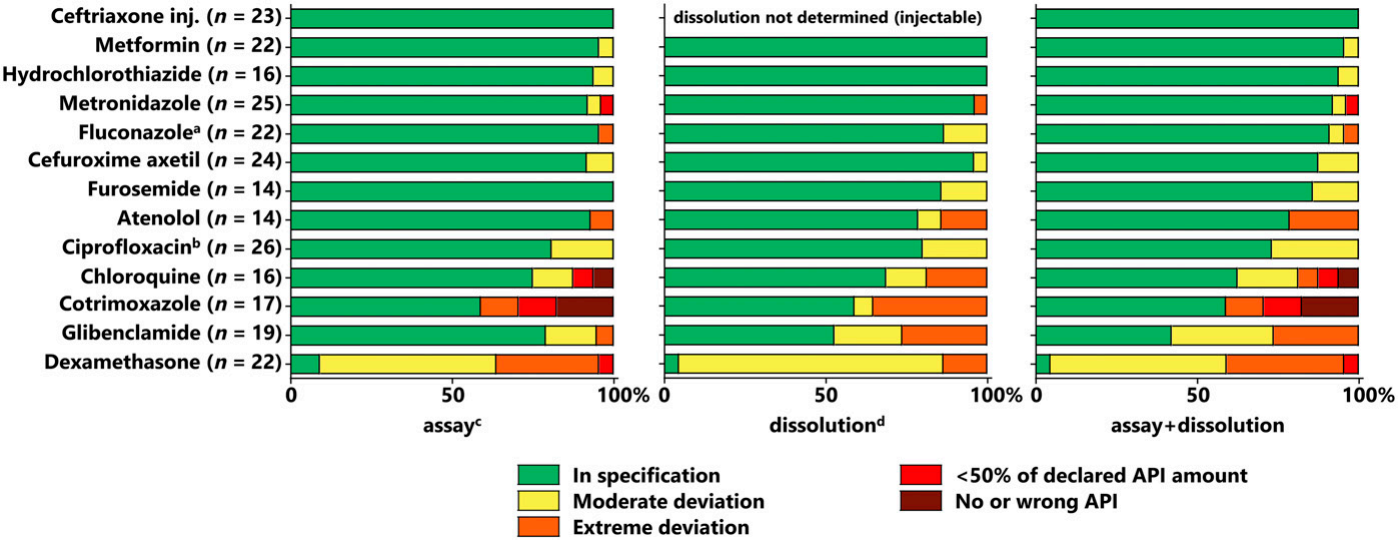
Results of compendial analysis for the investigated active pharmaceutical ingredients (APIs). ^**a**^Only 15 of 22 fluconazole samples were tested for dissolution, since 7 samples were collected as capsules and no dissolution testing method for fluconazole capsules is specified in the United States Pharmacopeia (USP). ^**b**^Only 25 of 26 ciprofloxacin samples were tested for dissolution, because not enough dosage units were available for one of the samples. ^**c**^For assay values, the following five categories were used^4^^,^^39^^,^^43^: 1) containing no or wrong APIs (i.e., “falsified”); 2) containing less than 50% of the declared API amount, without evidence that their low content was due to API degradation (i.e., “probably falsified”); 3) other samples deviating by more than 20% from the declared API amount (i.e., “extreme deviation”); 4) deviations from USP assay specifications by not more than 20% of the declared API amount (i.e., “moderate deviation”); 5) in specification. ^**d**^For dissolution values, average dissolution values lower than *Q* minus 25% were considered “extreme deviations”; dissolution values below USP specifications but not lower than *Q* minus 25% on average were considered “moderate deviations”.^43^ The categories “falsified” and “probably falsified” were not used, as these categories were based on assay results alone.

As summarized in Figure 5, the results were very different for the different investigated APIs. No or relatively few quality problems were found for ceftriaxone injections or for metformin, hydrochlorothiazide, metronidazole, and fluconazole tablets (or capsules). Serious quality problems were found for chloroquine and cotrimoxazole tablets; similar observations for the two latter APIs have been made in previous studies,^9^^,^^26^^,^^33^^,^^48^ for chloroquine especially after it had been (incorrectly) alleged to be effective in the treatment of COVID-19.^33^ Furthermore, more than half of the investigated glibenclamide samples were found to be OOS, mostly due to dissolution failures. Although shortcomings of glibenclamide tablets in dissolution testing have been reported previously,^11^^,^^49^ the extent of this problem observed in the present study was unexpected.

### Recall of substandard dexamethasone tablets in Nigeria.

Among all medicines investigated in this study, the most striking result was found for dexamethasone tablets (Figure 5). Out of 22 samples, 21 did not comply with pharmacopeial specifications, with 20 of these already failing in assay testing. As we had never found such a high proportion of failures before, this observation prompted us to reconfirm the accuracy of our analytical method by an interlaboratory comparison. Three of the dexamethasone samples were sent to the WHO-prequalified medicine quality control laboratory of the Mission for Essential Drugs and Supplies (MEDS) in Nairobi, Kenya, without communicating the already obtained analytical results. The samples were analyzed by MEDS for their API content according to the USP. The assay values determined by MEDS and by Tübingen University were in very good agreement: they deviated from the mean assay value for the respective sample on average by ±1.2%, with MEDS reporting for two samples a lower content and for one sample a higher content than that reported by Tübingen University.

According to the labels, the 22 dexamethasone samples had been produced by 14 different manufacturers: 10 from India, three from Nigeria, and one from China. Notably, the two samples that complied with assay specifications had been produced in Nigeria.

As shown in Figure 3A, seven of the dexamethasone samples failed the pharmacopeial specifications for assay only by a narrow margin. However, all of these seven samples failed the pharmacopeial specifications for dissolution very clearly: all of them showed dissolution values below 74%, and five of them showed dissolution values even below 65% (*Q* value, 80%).

Six of the dexamethasone samples were found to contain small amounts of the preservatives methylparaben and/or propylparaben. Although there is no clinical evidence of adverse effects in humans related to parabens, the European Medicines Agency states that the use of these substances should be avoided wherever possible.^50^

In view of the importance of the above-mentioned findings on dexamethasone tablets, they were communicated to the WHO on September 16, 2022, and subsequently by the WHO to the NAFDAC. Probably based on this report, the NAFDAC recalled these substandard dexamethasone products through a public alert on October 8, 2022,^51^ in a swift and decisive reaction.

### Results of compendial analysis for different manufacturers.

**Figure 6.**
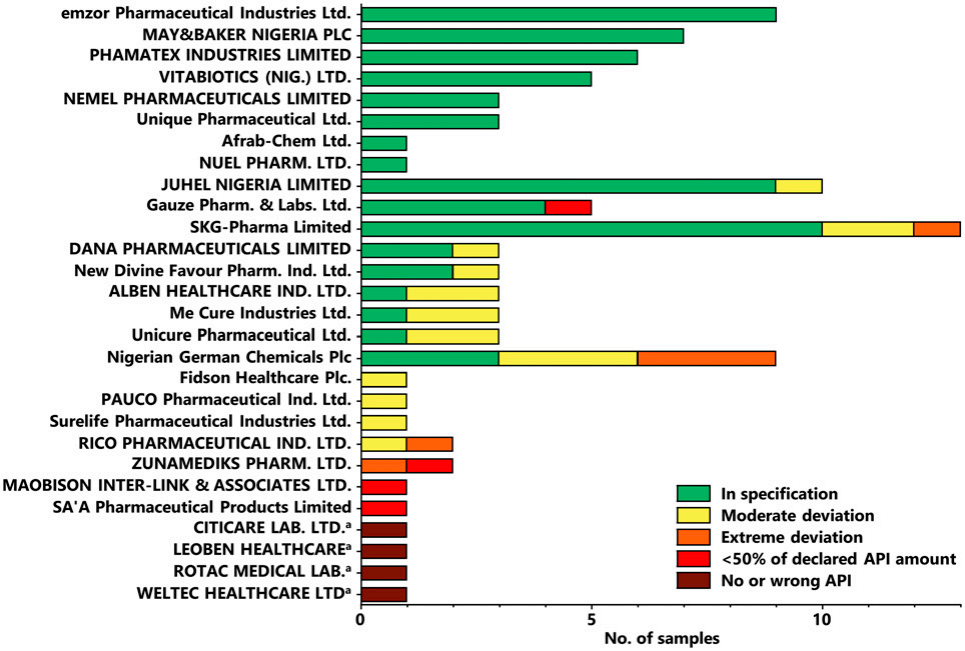
Results of compendial analysis for different stated manufacturers from Nigeria. See the legend for Figure 5 for definitions of quality categories. ^**a**^The names of the four stated manufacturers of falsified medicines could not be found by an internet search nor in Nigeria’s Registered Drug Product Database.^27^ Apparently, these four manufacturers do not exist.

Figure 6 shows the results of the compendial testing for the 28 stated manufacturers from Nigeria. For eight manufacturers (together representing 35 samples), all their investigated medicines complied with pharmacopeial specifications. On the other hand, for 11 stated manufacturers (together representing 13 samples), none of the tested samples complied with specifications. Therefore, careful manufacturer selection is important for quality assurance in medicine procurement, although larger numbers of samples from each manufacturer need to be investigated. The data in Figure 6 indicate a tendency that larger manufacturers, supplying a higher number of samples, provided medicines with better quality than manufacturers supplying only one single sample. An exception is the stated manufacturer Nigerian-German Chemicals Plc who supplied nine samples, but six of these (66.7%) failed pharmacopeial specifications.

As with Figure 6, Supplemental Figures S3, S4, and S5 show the results of compendial testing for the stated manufacturers from India, China, and other countries, respectively.

### Confirmation of NAFDAC registration status of the collected medicines.

For any medicine marketed in Nigeria, displaying a NAFDAC registration number on the packaging is an obligatory requirement.^52^ Among the 260 samples collected in this study, only 15 (representing six different APIs and 11 different brands) did not carry a NAFDAC registration number. All of them had been collected in markets. All of them were stated to be manufactured outside of Nigeria, or the manufacturer was not stated at all (only the marketing authorization holder). Twelve of these 15 samples complied with pharmacopeial specifications, and three showed moderate deviations. Therefore, according to the current WHO definitions,^2^^,^^42^ these products are not to be considered as falsified but as nonregistered medical products. In a similar medicine quality study in Malawi,^53^ only 61% of the collected medicine samples were registered by the national medicines regulatory authority (NMRA); in comparison, the proportion of samples carrying a NAFDAC registration number in the present study (93%) is remarkably high.

Two hundred forty-five samples collected in this study did carry a NAFDAC registration number. On the African continent, NAFDAC is among the leading NMRAs regarding the online provision of information on the registration status of medicines, through Nigeria’s Registered Drug Product Database, also called the NAFDAC Greenbook.^27^ For 96 samples, the registration numbers given on the product labels could be correctly verified in the NAFDAC Greenbook. The registration numbers stated on the product labels were, according to the NAFDAC Greenbook, given to products of different APIs and brands only for three collected samples. One of these three was a falsified cotrimoxazole sample, mentioned above. The other two were stated to represent the brand “Aphantix” (furosemide tablets; stated manufacturer, Mancare Pharmaceuticals Pvt. Ltd., India; stated NAFDAC registration number, 04-9146). Both had been collected in markets, and both complied with pharmacopeial specifications for assay and dissolution. It cannot be decided whether they carried an incorrect registration number simply because of an unintended mistake or had been produced and marketed with fraudulent intent.

For 146 samples, the registration number stated on the label could not be found in the NAFDAC Greenbook. It is possible that their registration status had expired and had yet to be renewed. On the other hand, we noticed that some of these registration numbers were added correctly during the time of our data analysis, possibly indicating ongoing work of the NAFDAC on the completion of that database. (In the present study, the final comparison of observed registration numbers to the database was carried out in September 2023.)

Of the 96 samples with a verifiable registration number, 18.8% were OOS (95% CI, 11.1–29.6%). In contrast, of the 149 samples with a nonverifiable or incorrect registration number, 30.2% were OOS (95% CI, 22.0–40.4%; *P* = 0.046). Although this difference is statistically significant, our data show that the presence of a verifiable NAFDAC registration number does not exclude the risk of that medicine being substandard or even extremely substandard, as shown in Figure 4. The percentages of samples with nonverifiable registration numbers were similar in products from licensed vendors (59.5%) and from markets (61.9%).

### MAS scheme.

**Figure 7.**
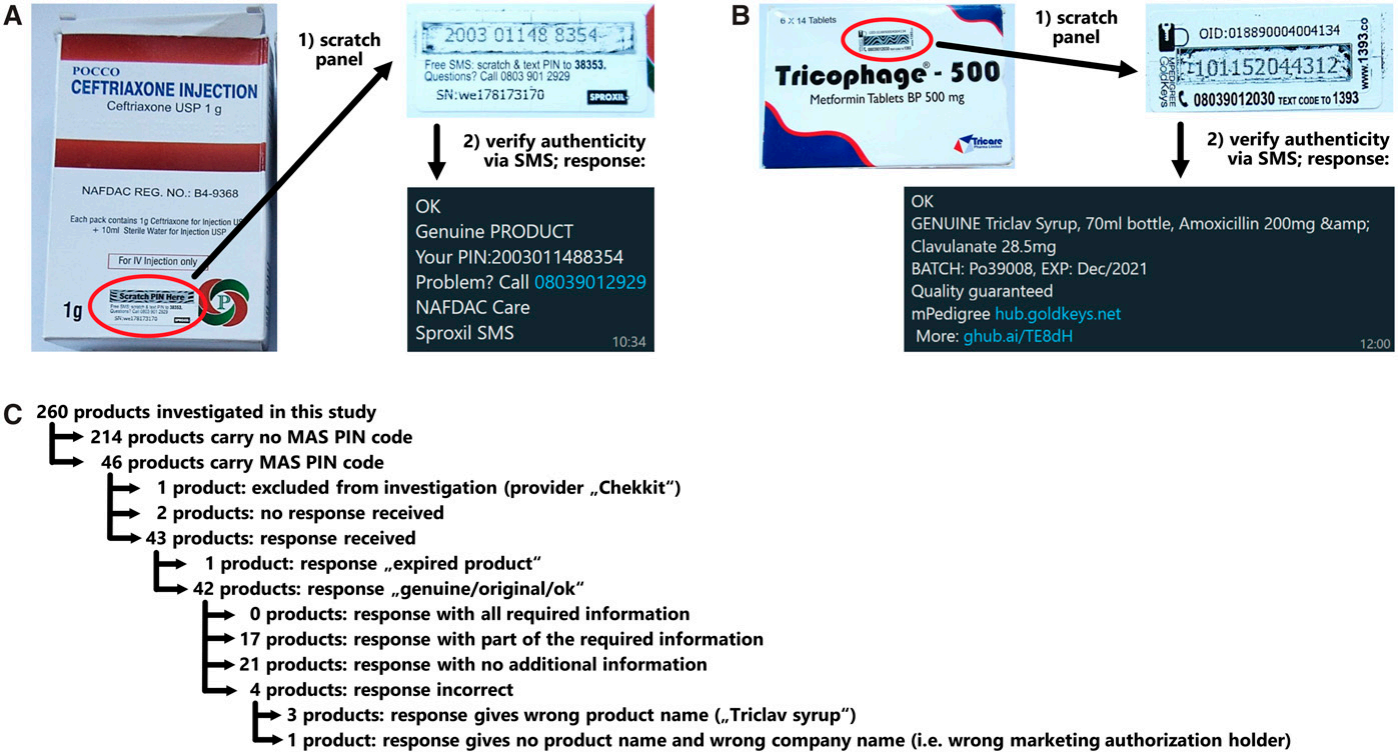
(**A**) SMS verification of a Mobile Authentication Service (MAS) PIN code resulting in a correct but incomplete response; see text for National Agency for Food and Drug Administration and Control (NAFDAC) requirements for a complete response. (**B**) SMS verification of a MAS PIN code resulting in an incorrect response (i.e., a wrong product name). (**C**) Summary of all results of MAS PIN code testing.

The NAFDAC took a pioneering step by instituting a medicine package serialization project called the Mobile Authentication Service (MAS) scheme, explained in detail in a NAFDAC guideline of 2018.^28^ For products of antiprotozoal and antibacterial APIs listed in that guideline, each medicine package manufactured in or imported into Nigeria is to be labeled by the marketing authorization holder (or by the manufacturer) with a unique PIN code (Figure 7A and B). The PIN is hidden by an opaque covering that can be scratched off by the consumer. The consumer can send this PIN toll-free via SMS to a telephone number displayed next to the PIN (Figure 7A and B) belonging to one of the five service providers contracted by the NAFDAC for this scheme.^28^ The consumer then receives an automatic response in the form of a text message (SMS). Besides the information about whether the sample is a genuine product, the message should contain at least the name, the NAFDAC registration number, the batch number and expiry date of the product, and a helpline telephone number for further information and for the reporting of nonverifiable products.^28^ This is a unique and interesting scheme, and the present study provided an opportunity to gather some data on its current functionality.

According to the NAFDAC guideline, six of the anti-infective APIs investigated in this study were expected to be included in the MAS scheme. As shown in Table 3, only for three of those APIs MAS PINs were indeed found, and in each case only for part of the respective samples. The overall coverage by the MAS scheme for samples of the six anti-infective APIs was 29.0% (Table 3). Several samples of fluconazole and metformin also carried MAS PINs, although these APIs are not mentioned in the guideline of 2018. Unexpectedly, by far most of the medicines carrying a MAS PIN had been manufactured abroad and hardly any in Nigeria (Table 3).

**Table 3:** Medicine samples carrying NAFDAC MAS scheme PIN codes

APIs	Samples Manufactured in Nigeria			Imported Samples			All Samples		
	Total (*n*)	With PIN		Total (*n*)	With PIN		Total (*n*)	With PIN	
		*n*	%		*n*	%		*n*	%
Listed in NAFDAC MAS guideline									
Chloroquine	16	0	0	0	0	–	16	0	0
Cotrimoxazole	17	0	0	0	0	–	17	0	0
Metronidazole	25	0	0	0	0	–	25	0	0
Ceftriaxone injection	0	0	–	23	7	30	23	7	30
Cefuroxime axetil	0	0	–	24	17	71	24	17	71
Ciprofloxacin	6	2	33	20	12	60	26	14	54
Total	64	2	3	67	36	54	131	38	29
Not listed in NAFDAC MAS guideline									
Fluconazole	2	0	0	20	2	10	22	2	9
Metformin	9	2	22	13	4	31	22	6	27
Grand total	75	4	5	100	42	42	175	46	26

MAS = Mobile Authentication Service; NAFDAC = National Agency for Food and Drug Administration and Control; PIN = personal identification number.

In total, out of the 260 samples collected in this study, 46 carried a MAS PIN (Table 3). Of those, 45 showed the contact numbers of the NAFDAC-approved service providers M-Pedigree (28 samples), Sproxil (15 samples), UBQ-t/Kezzler (one sample), and PharmaSecure (one sample). One further sample carried a PIN of the service provider Chekkit, which is not named in the NAFDAC guideline and was therefore excluded from further investigation. Compendial analysis showed that this sample complied with USP specifications.

None of the 45 investigated MAS PINs had been scratched free prior to the investigation in this study. They were now scratched free, and the PIN that appeared was forwarded to the respective service providers by SMS, using a mobile phone in Nigeria. The answers received are summarized in Figure 7C and are shown in full detail in Supplemental Table S3. Only for two samples was no response received. Among the 43 received responses, one stated (correctly) that the product in question was expired at the time of this testing (June 2023). All other 42 responses gave confirmation that the sample in question was a genuine product. Compendial analysis in this study had shown that 39 of these samples were in specification, and three (7.1%) showed moderate deviations.

Unexpectedly, not a single one of the SMS responses provided the complete obligatory information specified by the NAFDAC guideline.^28^ In 21 cases, the only information was the claim that the product was genuine, with no mention of the product name, NAFDAC registration number, or batch number or expiry date (see Figure 7A as example). Among the cases where at least some of the obligatory information was given, the information was incorrect in the case of four samples. Three products of the Indian manufacturer Baroque Pharmaceuticals Pvt. Ltd., representing cefuroxime axetil, metformin, and fluconazole tablets, respectively, were incorrectly stated to represent “Triclav Syrup” (Figure 7B), a brand of amoxicillin/clavulanic acid that is currently not listed in the NAFDAC Greenbook. For one further sample, the SMS response did not specify the product name but stated a marketing authorization holder that, however, was different from the one stated on the product label (Supplemental Table S3).

Among the 43 PINs for which SMS responses were received, the average response time was 38 seconds, the shortest for the provider M-Pedigree (14 seconds) and the longest for the provider Pharmasecure (79 seconds).

The NAFDAC guideline^28^ specifies that the MAS PINs must be for one-time use only. If the same PIN is sent to the service provider repeatedly, the expected answer is “PIN used,” which is important since otherwise a single valid PIN could be copied from a genuine package and be attached to multiple falsified medicine packages. In the present study, 16 of the above investigated MAS PINs were sent to the respective service provider for a second time. In eight cases, the correct response “PIN used” was received. However, in four cases the unmodified previous response was received, claiming that the product was genuine. In four further cases, no response at all was received within a predecided waiting time of 5 minutes.

As mentioned above, chemical analysis in the course of this study identified 9 out of the 260 samples as falsified or probably falsified. However, none of these nine products carried a MAS PIN code, and they could therefore not be examined for SMS responses within the MAS scheme.

## DISCUSSION

The key aim of the present study was to assist the FBCMF and similar stakeholders in Nigeria in their future decision-making and resource management for quality assurance in medicine procurement. For this purpose, selected types of medicines were purchased from different sources and analyzed for the content and dissolution of the APIs. Overall, 260 samples were investigated, and 25.4% of these were found to be OOS in assay, dissolution, or both. In comparison, a systematic review by the WHO in 2017,^2^ which summarized the results of 100 medicine quality studies, reported that the average prevalence of SF medicines in LMICs was 10.5% when all included studies were considered, and 15.6% when only studies using HPLC for analysis were considered (as with the present study) but not studies using less sensitive detection methods like the GPHF-Minilab.^10^

Our own group conducted a study in Cameroon and the DRC^11^ with analytical methodology very similar to that in the present investigation. Eight of the medicine types (APIs and formulations) included in that previous study were also included in the present study (Table 2), allowing a direct comparison of the results. When only these eight medicine types were considered, the percentages of samples with moderate deviations from USP specifications were very similar in the two studies (11.8% and 11.9%, respectively). However, the rate of samples with extreme deviations (including falsified and probably falsified medicines) in the present study in Nigeria was 10.5% (95% CI, 6.0–17.0%), three times higher than the rate of 3.1% (95% CI, 1.4–5.8%) observed in the study in Cameroon and the DRC (*P* = 0.001). Likewise, the rate of falsified and probably falsified samples among these eight medicine types was 3.9% (95% CI, 1.4–8.5%) in the present study, significantly higher than the rate of 0.3% (95% CI, 0.01–1.9%) observed in Cameroon and the DRC (*P* = 0.004). All the above comparisons, however, should be interpreted with caution, because the present study was not designed as a prevalence study with randomized selection of the sampling sites and was carried out only in a limited region of Nigeria.

Although the number of SF medicines detected in this study is concerning, it is markedly lower than frequently speculated in the lay press and in some scholarly publications.^54^^,^^55^ For example, in a survey among 541 health professionals in Nigeria, the proportion of “fake” medicines on the Nigerian market was estimated to be 49% on average.^56^ A Nigerian official of the National Drug Law Enforcement Agency was even quoted by the press as stating that in Nigeria, 70% of all the drugs on the market would be “fake.”^57^^,^^58^ A much lower percentage of falsified medicines was found in the present study, and this is in agreement with the results of previous scientific studies in Nigeria (see Introduction). More systematic research with appropriate methodology is desirable and could complement the present communications by the NAFDAC^57^ to curb unfounded, exaggerated speculations about the proportion of falsified and substandard medicines in Nigeria.

The sample collection for the present study was carried out during the COVID-19 pandemic. This pandemic resulted in a sudden increase in demand for medicines with alleged or true relevance for COVID-19 therapy, creating an opportunity for criminals to market falsified versions of such medicines.^59^ Indeed, falsified chloroquine tablets were found in the present study, similar to observations made previously in other African countries.^33^^,^^48^ Especially, and as a new observation, an extremely high rate of poor-quality dexamethasone tablets was detected, most of these imported from India and China. This finding calls for a thorough investigation of the quality of dexamethasone products in other countries as well. The swift recall of the substandard dexamethasone tablets by the NAFDAC^51^ confirms that effective structures are in place to implement such recalls in Nigeria.

In medicine quality studies in the past, anti-infective medicines tended to be overrepresented in comparison with medicines against NCDs.^2^ In this study, we found a slightly higher percentage of medicines against NCDs than against anti-infectives to be OOS, and similar findings were made in our previous study in Cameroon and the DRC.^11^ Mortality from NCDs is already high in sub-Saharan Africa and is still rising,^60^ and a more extensive inclusion of medicines against NCDs in future medicine quality studies is desirable.

Unexpectedly, and in contrast to findings from other countries,^11^^,^^61^ the proportions of OOS samples were found to be similar in markets and in licensed vendors in the present study. However, no clearly falsified medicines were found from licensed vendors. It should be noted that the “open drug markets”^36^^,^^54^ in Onitsha and Enugu are not simple roadside market stalls like in some neighboring African countries but are shops with a professional appearance, often very well stocked with many types of medicines. Our data show that simple adherence to licensed vendors is not sufficient to exclude substandard medicines from medicine procurement. As shown in Figure 1, the replacement of imported medicines by locally produced ones is also not sufficient, as the proportions of OOS samples are similar in medicines from Nigeria, India, and China.

The MAS scheme by the NAFDAC is currently intended to cover only the most important anti-infective medicines.^28^ However, even for these medicines, 71% of the samples collected in this study did not carry a MAS PIN code (65% in licensed vendors and 77% in markets). In addition, some technical shortcomings and mistakes have been observed, similar to previous reports.^62^ Therefore, the verification of MAS PIN codes appears to be of limited value in quality assurance at present.^62^^–^^65^

Preferential procurement of medicines prequalified by the WHO Medicine Prequalification Program^66^^,^^67^ would certainly be useful if such medicines were available and affordable in Nigeria. However, of the 13 types of medicines investigated in this study, only four are included in that program (ceftriaxone, ciprofloxacin, cotrimoxazole, and dexamethasone), and each of them only with a small number of commercial products. Therefore, none of the 260 samples collected in this study represented a WHO-prequalified product. A substantial expansion of this WHO program is probably necessary to make it useful for procurement agencies in Nigeria.

As part of its quality assurance, the FBCMF carries out visual inspection of procured medicines^68^ and chemical investigation using the GPHF-Minilab.^10^ Similar to previous studies,^26^ the present study found visual inspection to be a valuable tool for the identification of falsified medicines (see Results). Also, screening analysis with the GPHF-Minilab was found to reliably detect falsified medicines with no or wrong APIs (data not shown), as also reported in previous studies.^11^ However, the GPHF-Minilab detects medicines of insufficient amount or dissolution of the API only with low sensitivity,^11^ and simple, inexpensive screening technologies that can reliably detect substandard medicines do not yet exist.^25^ The FBCMF has access to compendial medicine quality analysis through its membership in the EPN, which includes the WHO-prequalified medicine quality control laboratory of MEDS, Kenya,^26^ but this can be used for only a limited number of samples. Therefore, a comprehensive quality assurance system is required, based on WHO’s Model Quality Assurance System for Procurement Agencies^69^ and including prequalification of products, manufacturers, and suppliers as a key component. Another key component is visual inspection of all procured medicines as well as laboratory analysis of selected medicines using both screening methods like the GPHF-Minilab and, as far as possible, compendial analysis as done in the present study. Collaborations within large networks like EPN may greatly facilitate such quality assurance in medicine procurement.

## CONCLUSION

Even in the presence of a strong and dedicated national medicine regulatory authority like the NAFDAC, which has recently reached WHO Maturity Level 3,^70^ ensuring the quality of medicines in Nigeria remains a challenge. Contrary to common belief, the largest problem is not the occurrence of falsified medicines sold with criminal intent under fake product and fake manufacturer names but the high prevalence of substandard medicines, arising from insufficient resources, insufficient competence, and insufficient diligence in their manufacturing. This applies equally to medicines manufactured in Nigeria and to imported ones. Accordingly, a solution of this problem cannot be found by law enforcement measures alone but requires an evolutionary approach including continuous training and supervision efforts and involving many stakeholders. One part of these efforts must be a systematic prequalification of products and manufacturers in medicine procurement by private, nongovernmental, and governmental organizations.

## Supplemental Materials

10.4269/ajtmh.23-0837 Supplemental Materials
