# Supplementary material for: Quality of Essential Medicines from Different Sources in Enugu and Anambra, Nigeria
Source: Am J Trop Med Hyg. 2024 May 14;111(1):179–95. doi: 10.4269/ajtmh.23-0837 (PMC11229646; doi:10.4269/ajtmh.23-0837)
Supplement: Supplemental Materials [file tpmd230837.SD1.pdf]

Supplementary Material for

Quality of Essential Medicines from Different Sources in Enugu and Anambra, Nigeria

Julia Gabel,[1] Micha Lächele,[1] Katharina Sander,[1] Gesa Gnegel,[1] Nkiru Sunny-Abarikwu,[2] Rita Ezinwanne Ohazulike,[2] Juliet Ngene,[2] Jane F. Chioke,[2] Christine Häfele-Abah,[3] Lutz Heide[1]*

[1]Pharmaceutical Institute, Eberhard Karls University Tuebingen, Tuebingen, Germany; [2]Faith-Based Central Medical Foundation (FBCMF), Enugu, Nigeria; [3]German Institute for Medical Mission (Difaem), Tuebingen, Germany

Contents:

	Page
**Supplementary Figure S1**. "Eden Fluconazole 150mg Capsules", carrying a misspelled "WHO" logo.	2
**Supplementary Figure S2**. Five medicine samples containing less than 50% of the stated API, therefore considered as probably falsified medicines.	3
**Supplementary Figure S3.** Results of compendial analysis for different stated manufacturers from India.	4
**Supplementary Figure S4.** Results of compendial analysis for different stated manufacturers from China.	5
**Supplementary Figure S5.** Results of compendial analysis for different stated manufacturers from further countries.	5
**Supplementary Table S1**. Specifications for assay and dissolution analysis for each investigated active pharmaceutical ingredient.	6
**Supplementary Table S2.** List of all samples investigated in this study with their stated manufacturer and quality assessment regarding assay and dissolution analysis.	7
**Supplementary Table S3.** Results of the testing of Mobile Authentication Service (MAS) codes.	13

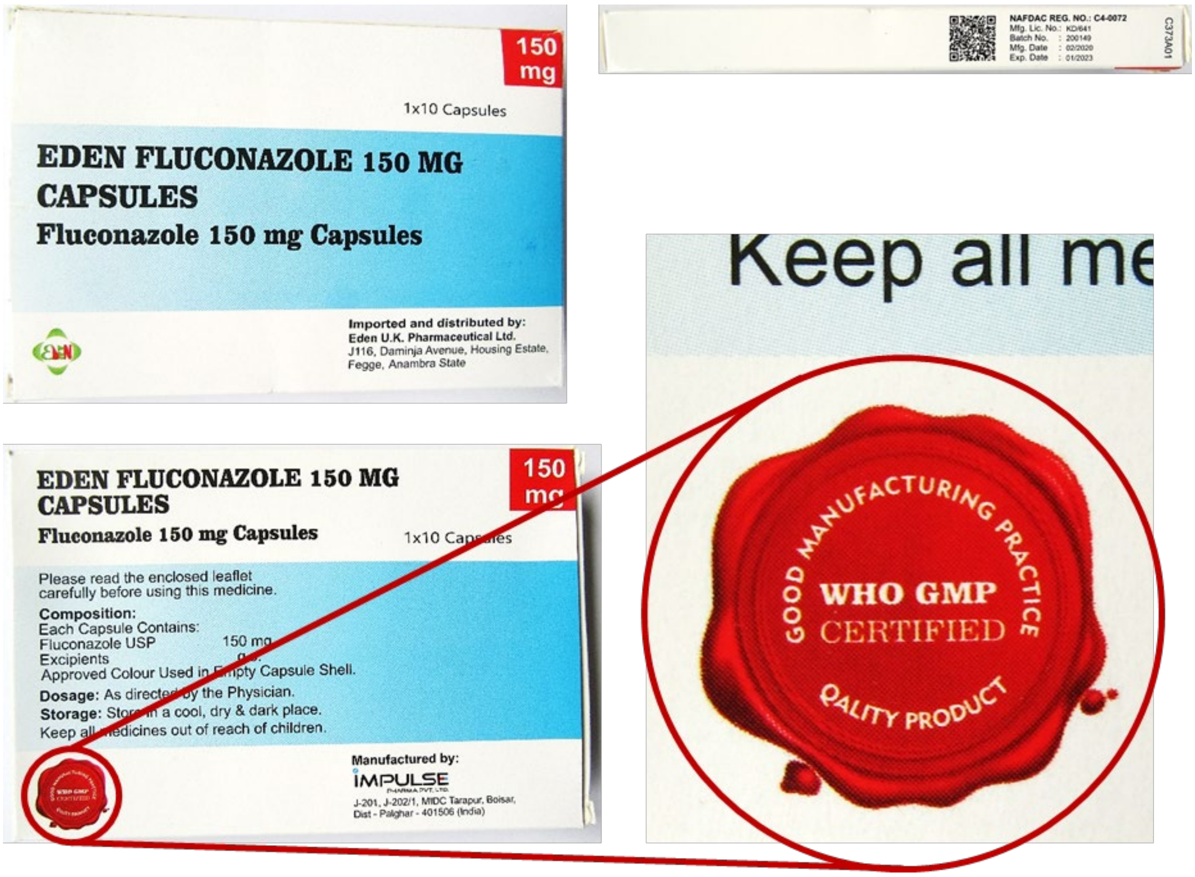

Supplementary Figure S1. "Eden Fluconazole 150mg Capsules", carrying a misspelled "WHO" logo. Note the spelling „QALITY" instead of „QUALITY". While WHO has published guidelines for the issuance of GMP certifications by national authorities, WHO itself does not issue such certificates. Nevertheless, the sample complied with USP specifications for the content of the API.

A) „SA'A QUINE" (chloroquine phosphate) by stated manufacturer SA'A Pharmaceutical Products Limited; 13.1% of stated API content

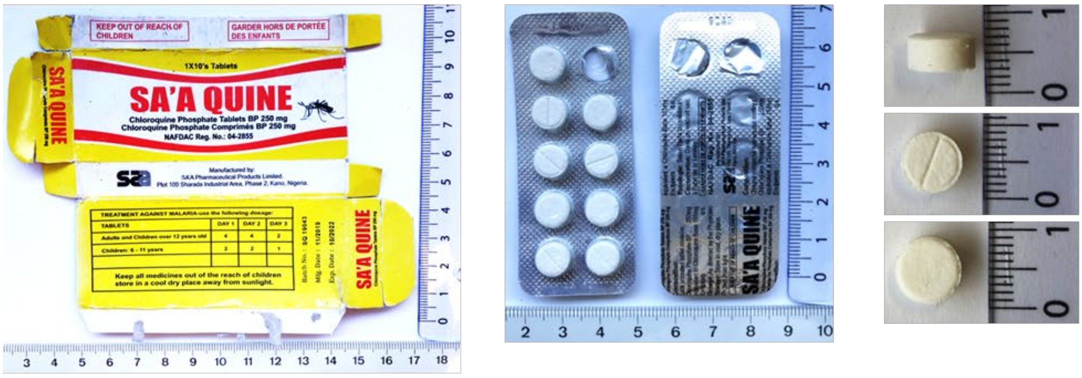

B) „POLETRIM" (sulfamethoxazole/ trimethoprim) by stated manufacturer MAOBISON INTER-LINK & ASSOCIATES LTD; 50.1%/22.4% of stated API content

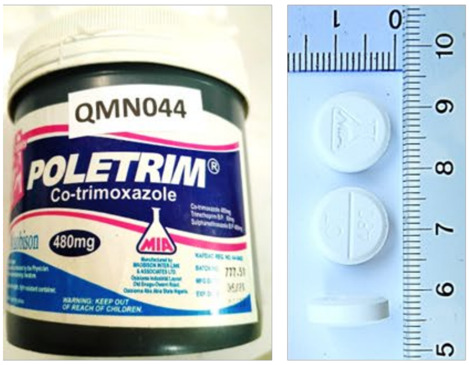

C) „ZIMATRIM"(sulfamethoxazole/ trimethoprim) by stated manufacturer Gauze Pharm. & Labs. Ltd.; 103.5%/23.9% of stated API content

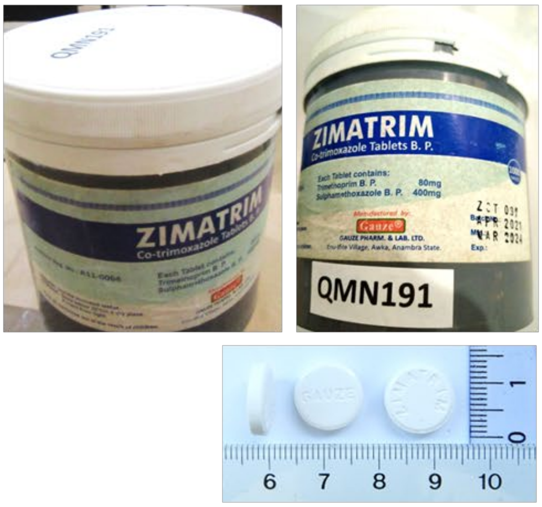

D) „ZUNAGYL" (metronidazole) by stated manufacturer ZUNAMEDIKS PHARM. LTD.; 48.4% of stated API content

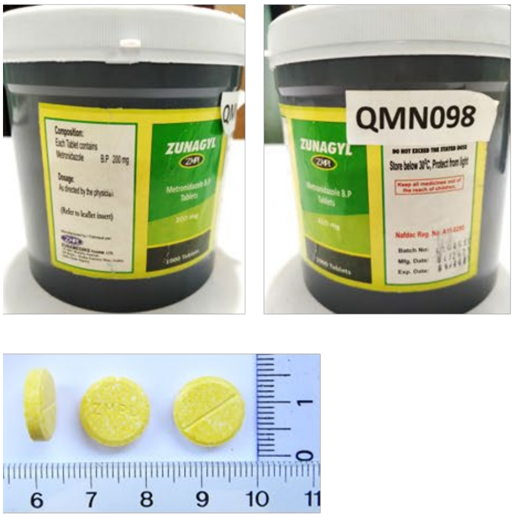

E) „Destrax" (dexamethasone) by stated manufacturer Jiangsu Pengyao Pharmaceuticals Co. Ltd.; 42.9% of stated API content

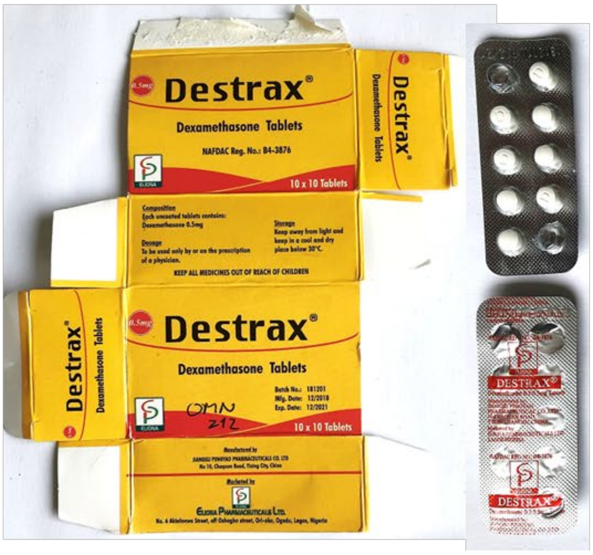

Supplementary Figure S2. Five medicine samples containing less than 50% of the stated API, without evidence that their low content was due to API degradation, therefore considered as probably falsified medicines.

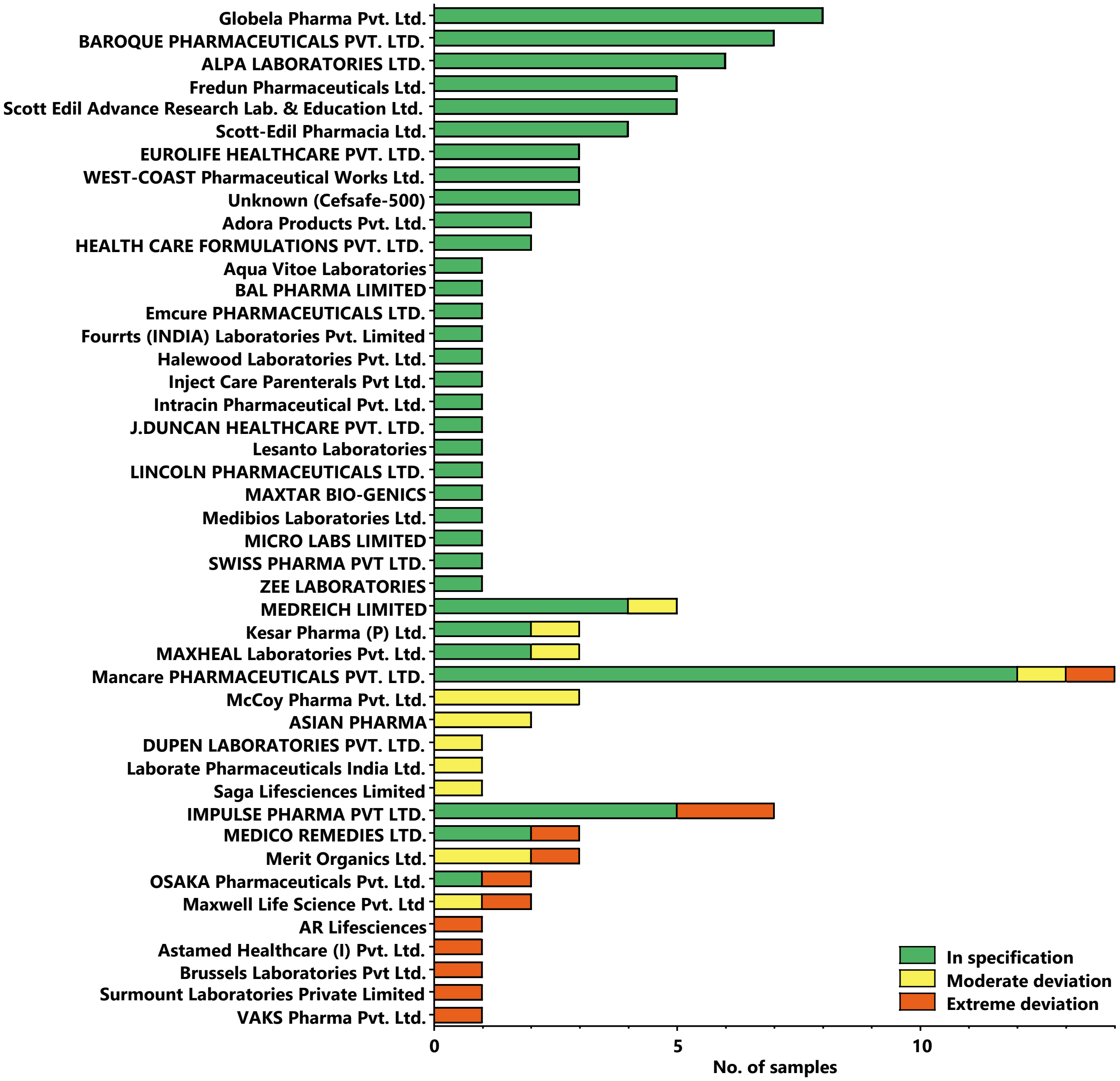

Supplementary Figure S3. Results of compendial analysis for different stated manufacturers from India.

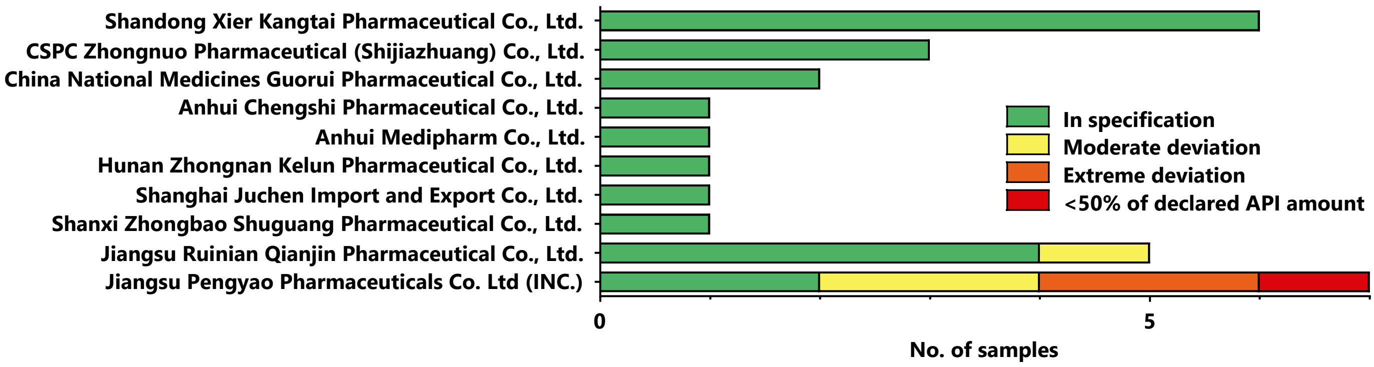

Supplementary Figure S4. Results of compendial analysis for different stated manufacturers from China.

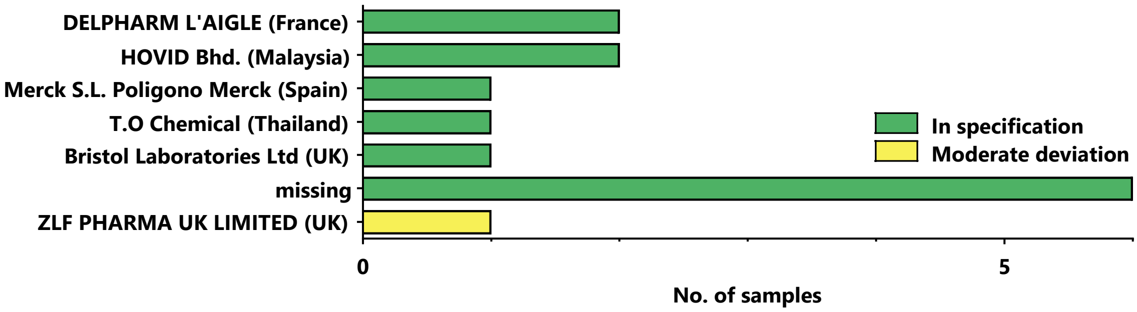

Supplementary Figure S5. Results of compendial analysis for different stated manufacturers from further countries.

Supplementary Table S1. Specifications for assay and dissolution analysis for each investigated active pharmaceutical ingredient.

Active pharmaceutical ingredient (API)	Dosage form tested	USP 42 specification for content of the API (=assay) [% of declared content)	USP 42 specification for dissolution of the API (Q value) [% of declared content]	Specification by Rahman et al. 2021[§] for stage S_1 dissolution testing	
				Average dissolution rate of n=3 units[#]	Minimum dissolution rate found in n=3 units[#]
Atenolol	tablet	90.0-110.0	≥80	≥86	≥82
Ceftriaxone sodium	powder for injection	90.0-115.0	-	-	-
Cefuroxime axetil	tablet	90.0-110.0	≥70*	≥76	≥72
Chloroquine phosphate and sulphate	tablet	93.0-107.0	≥75	≥81	≥77
Ciprofloxacin hydrochloride	tablet	90.0-110.0	≥80	≥86	≥82
Dexamethasone	tablet	90.0-110.0	≥80[&]	≥86	≥82
Fluconazole	tablet	90.0-110.0	≥75	≥81	≥77
Furosemide	tablet	90.0-110.0	≥80	≥86	≥82
Glibenclamide	tablet	90.0-110.0	≥70	≥76	≥72
Hydrochlorothiazide	tablet	90.0-110.0	≥60	≥66	≥62
Metformin hydrochloride	tablet	95.0-105.0	≥70	≥76	≥72
Metformin hydrochloride	extended-release tablet	90.0-110.0	≥85[$]	≥91	≥87
Metronidazole	tablet	90.0-110.0	≥85	≥91	≥87
Co-trimoxazole	tablet	93.0-107.0	≥70	≥76	≥72

[§] Rahman et al. 2021. Small-scale dissolution test screening tool to select potentially substandard and falsified (SF) medicines requiring full pharmacopoeial analysis. Sci Rep 11: 12145.

[#] If one or both of these limits were not met, stage S_2 testing was carried out according to the United States Pharmacopeia (USP).

* USP specification for dissolution after 45 min. USP specification for dissolution after 15 min: Q ≥50%.

[$] USP specification for dissolution after 10 hrs. USP specification for dissolution after 1hr: Q ≥20%; after 3 hrs: Q ≥45%.

[&] United States Pharmacopeia, 2021. Dexamethasone Tablets - Notice of Intent to Revise. Available at: https://www.uspnf.com/sites/default/files/usp_pdf/EN/USPNF/revisions/dexamethasone-tabs-pending-nitr-20210730.pdf. Accessed November 15, 2023.

Supplementary Table S2: List of all samples investigated in this study

Note: Many medicines were purchased not directly from their manufacturer but from other commercial sources. For those medicines it cannot be verified whether the manufacturers’ storage recommendations have been complied with from the time of manufacture until the time of sample collection. Changes in medicines quality may have occurred due to inappropriate transport and storage conditions, and therefore non-compliance with USP specifications is not necessarily due to substandard manufacturing or packaging. However, the results listed below reflect the quality in which health facilities, and ultimately patients, would receive these medicines.

* Co-trimoxazole comprises sulfamethoxazole and trimethoprim.

[§] Source A) Licensed pharmaceutical manufacturers and wholesalers ; Source B) Vendors in pharmaceutical markets of Onitsha and Enugu with unclear licensing status.

[#] For definitions of medicine quality refer to the main manuscript. USP 42 criteria was applied.

No.	Stated product name	Stated active pharmaceutical ingredient	Stated manufacturer	Stated manufacturing country	Source[§]	NAFDAC-Reg. No	Verification of NAFDAC-Reg. No possible in Nigeria’s Registered Drug Product Database (Sep. 2023)	Batchnumber	Applied method for quantification of dissolution testing	Assessment of assay results[#]	Assessment of dissolution results[#]	Assessement of assay AND dissolution results[#]	Sample ID
1	Harvad Cefuroxime tablets	Cefuroxime axetil	ALPA LABORATORIES LTD.	India	B	B4-7976	verified	TC0009	UV/Vis	in specification	in specification	in specification	QMN 001
2	Cefsafe-500	Cefuroxime axetil	missing	India	B	C4-0252	verified	HH108B21	UV/Vis	in specification	in specification	in specification	QMN 002
3	Oxispa 500	Cefuroxime axetil	Zee Laboratories	India	B	A4-4096	verified	ZET1408	UV/Vis	in specification	in specification	in specification	QMN 003
4	Cefuroxime Deno	Cefuroxime axetil	Scott-Edil Advance Research Laboratories & Education Ltd.	India	B	C4-0958	not found	1130Z007	UV/Vis	in specification	in specification	in specification	QMN 004
5	Ciprofloxacin 500 mg	Ciprofloxacin	Shanghai Juchen Import and Exports Co. Ltd.	China	B	missing	-	200203	HPLC	in specification	in specification	in specification	QMN 005
6	Ugolife Ciprofloxacin-500	Ciprofloxacin	Kesar Pharma (P) Ltd.	India	B	B4-7467	not found	T20466	HPLC	in specification	in specification	in specification	QMN 006
7	Biocipro	Ciprofloxacin	McCoy Pharma Pvt. Ltd.	India	B	B4-2452	not found	MP9422	HPLC	moderate deviation	in specification	moderate deviation	QMN 007
8	Ciprofloxacin 500	Ciprofloxacin	Jiangsu Pengyao Pharmaceutical Co. Ltd. (INC.)	China	B	B4-5947	verified	201257	HPLC	in specification	in specification	in specification	QMN 008
9	Biophage 500	Metformin	SKG-Pharma Limited	Nigeria	B	A4-6597	verified	2126	HPLC	in specification	in specification	in specification	QMN 009
10	Gluformin	Metformin	Nigerian-German Chemicals Plc	Nigeria	B	04-6426	verified	FPB080321	HPLC	in specification	in specification	in specification	QMN 010
11	Metformin	Metformin	Fredun Pharmaceuticals Ltd.	India	B	B4-0944	not found	FK0002	HPLC	in specification	in specification	in specification	QMN 011
12	Transglobe's Metformin	Metformin	EUROLIFE HEALTHCARE PVT. LTD.	India	B	A4-4274	not found	TMN005	HPLC	in specification	in specification	in specification	QMN 012
13	AD-Fluconazole	Fluconazole	Globela Pharma Pvt. Ltd.	India	B	B4-1543	not found	20GT047	HPLC	in specification	in specification	in specification	QMN 013
14	Nkoyo Fluconazole	Fluconazole	Mancare PHARMACEUTICALS PVT. LTD.	India	B	A4-0421	not found	TUL45	HPLC	in specification	in specification	in specification	QMN 014
15	C Cotrim - 480	Co-trimoxazole*	CITICARE LAB. LTD.	Nigeria	B	04-2872	not found	018	HPLC	falsified	extreme deviation	falsified	QMN 015
16	Emtrim	Co-trimoxazole*	emzor Pharmaceutical Industries Ltd.	Nigeria	B	04-0267	verified	R0371SA	HPLC	in specification	in specification	in specification	QMN 016
17	Emgyl	Metronidazole	emzor Pharmaceutical Industries Ltd.	Nigeria	B	04-0412	not found	2557A	HPLC	in specification	in specification	in specification	QMN 017
18	Nemegyl	Metronidazole	NEMEL PHARMACEUTICALS LIMITED	Nigeria	B	04-5326	not found	11B	HPLC	in specification	in specification	in specification	QMN 018
19	Albegyl	Metronidazole	ALBEN HEALTHCARE IND. LTD.	Nigeria	B	A4-7707	verified	043	HPLC	in specification	in specification	in specification	QMN 019
20	Emgyl	Metronidazole	emzor Pharmaceutical Industries Ltd.	Nigeria	B	04-0412	not found	S0156SA	HPLC	in specification	in specification	in specification	QMN 020
21	New Divine Frusemide	Furosemide	New Divine Favour Pharmaceutical Industries Ltd.	Nigeria	B	A4-8776	not found	0029	UV/Vis	in specification	in specification	in specification	QMN 021
22	Lasimac	Furosemide	Mancare PHARMACEUTICALS PVT. LTD.	India	B	B4-6708	verified	TUK07	UV/Vis	in specification	in specification	in specification	QMN 022
23	Aphantix	Furosemide	Mancare PHARMACEUTICALS PVT. LTD.	India	B	04-9146	product of different API and brand registered under this Reg. No.	TVK32	UV/Vis	in specification	in specification	in specification	QMN 023
24	Nkoyosix	Furosemide	Mancare PHARMACEUTICALS PVT. LTD.	India	B	A4-9179	verified	TUL35	UV/Vis	in specification	in specification	in specification	QMN 024
25	Chloroquine	Chloroquine	emzor Pharmaceutical Industries Ltd.	Nigeria	B	04-1218	not found	1817Z	HPLC; UV/Vis	in specification	in specification	in specification	QMN 025
26	Quimal	Chloroquine	DANA PHARMACEUTICALS LIMITED	Nigeria	B	04-1785	verified	QT145	HPLC; UV/Vis	in specification	in specification	in specification	QMN 026
27	Sa'a Quine	Chloroquine	SA'A Pharmaceutical Products Limited	Nigeria	B	04-2855	not found	SQ19043	HPLC; UV/Vis	probably falsified	extreme deviation	probably falsified	QMN 027
28	Dexacure	Dexamethasone	Unicure Pharmaceutical Ltd.	Nigeria	B	A4-7117	verified	201101	HPLC	moderate deviation	moderate deviation	moderate deviation	QMN 028
29	Rich Dexa	Dexamethasone	Brussels Laboratories Pvt Ltd.	India	B	B4-5389	not found	Z19003	HPLC	extreme deviation	extreme deviation	extreme deviation	QMN 029
30	Dexamethasone 0.5	Dexamethasone	Surmount Laboratories Private Limited	India	B	B4-9983	not found	TD9003	HPLC	extreme deviation	moderate deviation	extreme deviation	QMN 030
31	Zanelb	Dexamethasone	VAKS Pharma Pvt. Ltd.	India	B	B4-8496	verified	V20169	HPLC	extreme deviation	moderate deviation	extreme deviation	QMN 031
32	Glanil	Glibenclamide	Nigerian-German Chemicals Plc	Nigeria	B	04-2450	not found	FPA070221	HPLC	in specification	extreme deviation	extreme deviation	QMN 032
33	Gliben-J	Glibenclamide	JUHEL NIGERIA LIMITED	Nigeria	B	04-5735	not found	0041	HPLC	in specification	in specification	in specification	QMN 033
34	Tionil	Glibenclamide	Merit Organics Ltd.	India	B	B4-7811	not found	T32002	HPLC	extreme deviation	extreme deviation	extreme deviation	QMN 034

35	Redrex 25	Hydrochlorothiazide	WEST-COAST Pharmaceutical Works Ltd.	India	B	unknown	-	WG19285	HPLC	in specification	in specification	in specification	QMN 035
36	Hydrex	Hydrochlorothiazide	JUHEL NIGERIA LIMITED	Nigeria	B	A4-1209	verified	0139	HPLC	in specification	in specification	in specification	QMN 036
37	Eden Atenolol	Atenolol	IMPULSE PHARMA PVT LTD.	India	B	B4-6760	not found	200164	HPLC	in specification	in specification	in specification	QMN 037
38	Atenolol	Atenolol	ALPA LABORATORIES LTD.	India	B	B4-8238	verified	TE0184	HPLC	in specification	in specification	in specification	QMN 038
39	Chupet Ceftriaxone Sodiun	Ceftriaxone	Hunan Zhongnan Kelun Pharmaceutical Co., Ltd.	China	B	A4-9142	verified	200303	-	in specification	-	in specification	QMN 039
40	Pocco Ceftriaxone Injectior	Ceftriaxone	Scott-Edil Advance Research Laboratories & Education Ltd.	India	B	B4-9368	verified	1340Z157	-	in specification	-	in specification	QMN 040
41	Rebok Ceftriaxone Sodium	Ceftriaxone	China National Medicines Guorui Pharmaceutical Co., Ltd.	China	B	A4-5327	not found	200946	-	in specification	-	in specification	QMN 041
42	Macephin	Ceftriaxone	Shandong Xier Kangtai Pharm Co. Ltd	China	B	B4-5559	not found	201221	-	in specification	-	in specification	QMN 042
43	Chloro	Chloroquine	LEOBEN HEALTHCARE	Nigeria	B	04-4166	not found	0432	HPLC; UV/Vis	falsified	extreme deviation	falsified	QMN 043
44	Poletrim	Co-trimoxazole*	MAOBISON INTER-LINK & ASSOCIATES LTD.	Nigeria	B	A4-8482	not found	777.51	HPLC	probably falsified	extreme deviation	probably falsified	QMN 044
45	Rotrim	Co-trimoxazole*	ROTAC MEDICAL LAB.	Nigeria	B	04-5745	product of different API and brand registered under this Reg. No.	RML478	HPLC	falsified	extreme deviation	falsified	QMN 045
46	Weltrim	Co-trimoxazole*	WELTEC HEALTHCARE LTD	Nigeria	B	04-5245	not found	NHL-1001	HPLC	falsified	extreme deviation	falsified	QMN 046
47	Hydrochlorothiazide	Hydrochlorothiazide	missing	missing	B	missing	-	AE-19118	HPLC	in specification	in specification	in specification	QMN 047
48	Redrex 25	Hydrochlorothiazide	WEST-COAST Pharmaceutical Works Ltd.	India	B	B4-9818	verified	WG20005	HPLC	in specification	in specification	in specification	QMN 048
49	Esidrex	Hydrochlorothiazide	DELPHARM L'AIGLE	France	B	missing	-	2OFA301	HPLC	in specification	in specification	in specification	QMN 049 (1/2)
50	Johnbee Fluconazole Table	Fluconazole	Scott-Edil Pharmacia Ltd.	India	B	B4-6796	not found	XT9L037	HPLC	in specification	in specification	in specification	QMN 050
51	Fungiban	Fluconazole	Mancare PHARMACEUTICALS PVT. LTD.	India	B	A4-8168	verified	TUE113	HPLC	extreme deviation	moderate deviation	extreme deviation	QMN 051
52	Eden Atenolol	Atenolol	IMPULSE PHARMA PVT LTD.	India	B	B4-6760	not found	200164	HPLC	in specification	in specification	in specification	QMN 052
53	Emgyl	Metronidazole	emzor Pharmaceutical Industries Ltd.	Nigeria	B	04-0412	not found	S0156SA	HPLC	in specification	in specification	in specification	QMN 053
54	Cisepro-500	Ciprofloxacin	Fredun Pharmaceuticals Ltd.	India	B	B4-6330	not found	AA0036	HPLC	in specification	in specification	in specification	QMN 054
55	G.Ossy Ceftriaxone	Ceftriaxone	Shandong Xier Kangtai Pharm Co. Ltd	China	B	B4-4640	not found	201216	-	in specification	-	in specification	QMN 055
56	Dexacure	Dexamethasone	Unicure Pharmaceutical Ltd.	Nigeria	B	A4-7117	verified	210401	HPLC	in specification	in specification	in specification	QMN 056
57	Diatab	Glibenclamide	MAY&BAKER NIGERIA PLC	Nigeria	B	04-7837	verified	A202435	HPLC	in specification	in specification	in specification	QMN 057
58	Glanil	Glibenclamide	Nigerian-German Chemicals Plc	Nigeria	B	04-2450	not found	FPD070421	HPLC	in specification	extreme deviation	extreme deviation	QMN 058
59	Sivophage	Metformin	Globela Pharma Pvt. Ltd.	India	B	B4-0684	not found	GT20258	HPLC	in specification	in specification	in specification	QMN 059
60	Gluformin	Metformin	Nigerian-German Chemicals Plc	Nigeria	B	04-6426	verified	FPD080121	HPLC	moderate deviation	in specification	moderate deviation	QMN 060
61	Atenolol	Atenolol	missing	missing	B	missing	-	BPQ170002	HPLC	in specification	in specification	in specification	QMN 061
62	Latrim 480	Co-trimoxazole*	Me Cure Industries Ltd.	Nigeria	B	04-4483	verified	CT.1146	HPLC	in specification	in specification	in specification	QMN 062
63	Metrozol	Metronidazole	VITABIOTICS (NIG.) LTD.	Nigeria	B	A4-6028	not found	T21521	HPLC	in specification	in specification	in specification	QMN 063
64	Esodrex 25 mg	Hydrochlorothiazide	Anhui Medipharm Co. Ltd	China	B	missing	-	190329	HPLC	in specification	in specification	in specification	QMN 064
65	Johnbee Fluconazole Table	Fluconazole	Scott-Edil Pharmacia Ltd.	India	B	B4-6796	not found	XT9L037	HPLC	in specification	in specification	in specification	QMN 065
66	Xymatyl 500	Cefuroxime axetil	Adora Products Pvt. Ltd.	India	B	B4-5993	not found	HG303L20	UV/Vis	in specification	in specification	in specification	QMN 066
67	Cefurite	Cefuroxime axetil	ASIAN PHARMA	India	B	missing	-	AT11721	UV/Vis	moderate deviation	in specification	moderate deviation	QMN 067
68	Lasimac	Furosemide	Mancare PHARMACEUTICALS PVT. LTD.	India	B	B4-6708	verified	TUK03	UV/Vis	in specification	in specification	in specification	QMN 068
69	Glulife-500 mg	Metformin	Jiangsu Ruinian Qianjin Pharmaceutical Co. Ltd.	China	B	A4-6354	not found	200310	HPLC	in specification	in specification	in specification	QMN 069
70	Me cure Dexamethasone	Dexamethasone	Me Cure Industries Ltd.	Nigeria	B	A4-0201	verified	DC.319	HPLC	moderate deviation	moderate deviation	moderate deviation	QMN 070
71	Dexacure	Dexamethasone	Unicure Pharmaceutical Ltd.	Nigeria	B	A4-7117	verified	210201	HPLC	moderate deviation	moderate deviation	moderate deviation	QMN 071
72	Dexacortin	Dexamethasone	Maxwell Life Science Pvt. Ltd.	India	B	04-2446	not found	ET9514	HPLC	extreme deviation	moderate deviation	extreme deviation	QMN 072
73	Xasten	Dexamethasone	Jiangsu Pengyao Pharmaceutical Co. Ltd. (INC.)	China	B	04-6822	verified	200925	HPLC	moderate deviation	moderate deviation	moderate deviation	QMN 073
74	Frusemide tablets shree ax	Furosemide	Mancare PHARMACEUTICALS PVT. LTD.	India	B	A4-0604	not found	TUK16	UV/Vis	in specification	in specification	in specification	QMN 074
75	Aphantix	Furosemide	Mancare PHARMACEUTICALS PVT. LTD.	India	B	04-9146	product of different API and brand registered under this Reg. No.	TVK28	UV/Vis	in specification	in specification	in specification	QMN 075
76	Lasimac	Furosemide	Mancare PHARMACEUTICALS PVT. LTD.	India	B	B4-6708	verified	TUK02	UV/Vis	in specification	in specification	in specification	QMN 076
77	Flucoxiom-150	Fluconazole	Lesanto Laboratories	India	B	C4-1024	not found	L718002	HPLC	in specification	not tested (capsule)	in specification	QMN 077
78	Fluconazole 150 mg	Fluconazole	Globela Pharma Pvt. Ltd.	India	B	B4-7568	not found	GT20110	HPLC	in specification	in specification	in specification	QMN 078
79	Nkoyo Fluconazole	Fluconazole	Mancare PHARMACEUTICALS PVT. LTD.	India	B	A4-0421	not found	TUL43	HPLC	in specification	in specification	in specification	QMN 079
80	Roxirite	Cefuroxime axetil	HEALTH CARE FORMULATIONS PVT. LTD.	India	B	C4-1189	verified	T-220	UV/Vis	in specification	in specification	in specification	QMN 080

81	Oxispa 500	Cefuroxime axetil	MEDICO REMEDIES LTD.	India	B	B4-6770	not found	OXS001	UV/Vis	in specification	in specification	in specification	QMN 081
82	Harvad Cefuroxime tablets	Cefuroxime axetil	ALPA LABORATORIES LTD.	India	B	B4-7976	verified	TC0011	UV/Vis	in specification	in specification	in specification	QMN 082
83	Ibu Ciprofloxacin - 500	Ciprofloxacin	Kesar Pharma (P) Ltd.	India	B	C4-0487	not found	T21001	HPLC	moderate deviation	in specification	moderate deviation	QMN 083
84	Osworth Metformin	Metformin	MAY&BAKER NIGERIA PLC	Nigeria	B	A4-4310	verified	AC20039	HPLC	in specification	in specification	in specification	QMN 084
85	Ciprofloxacin	Ciprofloxacin	Fredun Pharmaceuticals Ltd.	India	A	B4-1176	verified	FC0020	HPLC	in specification	in specification	in specification	QMN 085
86	Metformin	Metformin	Fredun Pharmaceuticals Ltd.	India	A	B4-0944	not found	FK0003	HPLC	in specification	in specification	in specification	QMN 086
87	Quimal	Chloroquine	DANA PHARMACEUTICALS LIMITED	Nigeria	B	04-1785	verified	QT145	HPLC; UV/Vis	moderate deviation	in specification	moderate deviation	QMN 087
88	Albequine	Chloroquine	ALBEN HEALTHCARE IND. LTD.	Nigeria	B	B4-0086	not found	017	HPLC; UV/Vis	in specification	moderate deviation	moderate deviation	QMN 088
89	Softhealth Ciprofloxcin tab	Ciprofloxacin	Halewood Laboratories Pvt. Ltd.	India	B	B4-6819	not found	HV1003	HPLC	in specification	in specification	in specification	QMN 089
90	Cipro-500	Ciprofloxacin	NUEL PHARM. LTD.	Nigeria	B	A4-9505	not found	0621NCPO2	HPLC	in specification	in specification	in specification	QMN 090
91	Ceftriaxone injection	Ceftriaxone	Anhui Chengshi Pharmaceutical Co. Ltd.	China	B	B4-7640	not found	303201201	-	in specification	-	in specification	QMN 091
92	Atenolol	Atenolol	missing	missing	B	missing	-	K4548002	HPLC	in specification	in specification	in specification	QMN 092
93	Atenolol	Atenolol	ALPA LABORATORIES LTD.	India	B	B4-8238	verified	TE0184	HPLC	in specification	in specification	in specification	QMN 093
94	New Divine Frusemide	Furosemide	New Divine Favour Pharmaceutical Industries Ltd.	Nigeria	B	A4-8776	not found	0029	UV/Vis	in specification	in specification	in specification	QMN 094
95	Furosemide	Furosemide	Bristol Laboratories Ltd	UK	B	missing	-	AUC240012	UV/Vis	in specification	in specification	in specification	QMN 095
96	Eden Atenolol	Atenolol	IMPULSE PHARMA PVT LTD.	India	B	B4-6760	not found	200165	HPLC	in specification	in specification	in specification	QMN 096
97	Zuntrim	Co-trimoxazole*	ZUNAMEDIKS PHARM. LTD.	Nigeria	B	A11-0988	verified	RT0003C	HPLC	extreme deviation	moderate deviation	extreme deviation	QMN 097
98	Zunagyl	Metronidazole	ZUNAMEDIKS PHARM. LTD.	Nigeria	B	A11-0295	verified	GT0063D	HPLC	probably falsified	extreme deviation	probably falsified	QMN 098
99	Elcexone	Ceftriaxone	Inject Care Parenterals Pvt Ltd.	India	B	B4-7427	not found	IC21263004	-	in specification	-	in specification	QMN 099
100	Derm Ceftriaxone 1G Injec	Ceftriaxone	Shandong Xier Kangtai Pharm Co. Ltd	China	B	B4-5844	not found	201172	-	in specification	-	in specification	QMN 100
101	Rebok Ceftriaxone Sodium	Ceftriaxone	China National Medicines Guorui Pharmaceutical Co., Ltd.	China	B	A4-5327	not found	200235	-	in specification	-	in specification	QMN 101
102	Macephin	Ceftriaxone	Shandong Xier Kangtai Pharm Co. Ltd	China	B	B4-5559	not found	201221	-	in specification	-	in specification	QMN 102
103	Betafil	Atenolol	Fourrts (India) Laboratories Pvt. Limited	India	B	04-8111	verified	H0410	HPLC	in specification	in specification	in specification	QMN 103
104	Atenolol	Atenolol	missing	missing	B	missing	-	BPQ179021	HPLC	in specification	in specification	in specification	QMN 104
105	Emgyl	Metronidazole	emzor Pharmaceutical Industries Ltd.	Nigeria	B	04-0412	not found	338A	HPLC	in specification	in specification	in specification	QMN 105
106	Loxagyl 200	Metronidazole	MAY&BAKER NIGERIA PLC	Nigeria	B	04-0283	not found	A182113	HPLC	in specification	in specification	in specification	QMN 106
107	Diamet	Metformin	MAY&BAKER NIGERIA PLC	Nigeria	B	04-7945	not found	A210792	HPLC	in specification	in specification	in specification	QMN 107
108	Tricophage - 500	Metformin	BAROQUE PHARMACEUTICALS PVT. LTD.	India	B	B4-2429	not found	G039008	HPLC	in specification	in specification	in specification	QMN 108
109	Roxirite	Cefuroxime axetil	HEALTH CARE FORMULATIONS PVT. LTD.	India	B	C4-1189	verified	T-218	UV/Vis	in specification	in specification	in specification	QMN 109
110	Cefurite	Cefuroxime axetil	ASIAN PHARMA	India	B	missing	-	AT11721	UV/Vis	moderate deviation	in specification	moderate deviation	QMN 110
111	Harvad Cefuroxime tablets	Cefuroxime axetil	ALPA LABORATORIES LTD.	India	B	B4-7976	verified	TC0011	UV/Vis	in specification	in specification	in specification	QMN 111
112	Cefuroxime Deno	Cefuroxime axetil	Scott-Edil Advance Research Laboratories & Education Ltd.	India	B	C4-0958	not found	1130Z007	UV/Vis	in specification	in specification	in specification	QMN 112
113	Emtrim	Co-trimoxazole*	emzor Pharmaceutical Industries Ltd.	Nigeria	B	04-0267	verified	S0412SA	HPLC	in specification	in specification	in specification	QMN 113
114	Tionil	Glibenclamide	Merit Organics Ltd.	India	B	B4-7811	not found	T32002	HPLC	in specification	moderate deviation	moderate deviation	QMN 114
115	Glibenclamide	Glibenclamide	ZLF PHARMA UK LIMITED	UK	B	missing	-	190201	HPLC	in specification	moderate deviation	moderate deviation	QMN 115
116	Glibenclamide	Glibenclamide	MEDICO REMEDIES LTD.	India	B	B4-8197	verified	GIB903	HPLC	moderate deviation	extreme deviation	extreme deviation	QMN 116
117	Transglobe	Glibenclamide	OSAKA Pharmaceuticals Pvt. Ltd.	India	B	unknown	-	OS20032	HPLC	in specification	in specification	in specification	QMN 117
118	Redrex 25	Hydrochlorothiazide	WEST-COAST Pharmaceutical Works Ltd.	India	B	B4-9818	verified	WG20004	HPLC	in specification	in specification	in specification	QMN 118
119	Hydrochlorothiazide	Hydrochlorothiazide	missing	missing	B	missing	-	AE-19118	HPLC	in specification	in specification	in specification	QMN 119
120	Hydrochlorothiazide	Hydrochlorothiazide	missing	missing	B	missing	-	D200272	HPLC	in specification	in specification	in specification	QMN 120
121	HCTZ 25	Hydrochlorothiazide	T.O Chemical	Thailand	B	missing	-	S102021	HPLC	in specification	in specification	in specification	QMN 121
122	Chloroquine	Chloroquine	emzor Pharmaceutical Industries Ltd.	Nigeria	B	04-1218	not found	1840Z	HPLC; UV/Vis	in specification	in specification	in specification	QMN 122
123	Dr. Meyer's Maxiquine	Chloroquine	VITABIOTICS (NIG.) LTD.	Nigeria	B	A11-0393	not found	T61220	HPLC; UV/Vis	in specification	in specification	in specification	QMN 123
124	Quimal	Chloroquine	DANA PHARMACEUTICALS LIMITED	Nigeria	B	04-1785	verified	QT145	HPLC; UV/Vis	in specification	in specification	in specification	QMN 124
125	Albequine	Chloroquine	ALBEN HEALTHCARE IND. LTD.	Nigeria	B	B4-0086	not found	017	HPLC; UV/Vis	moderate deviation	moderate deviation	moderate deviation	QMN 125
126	Gluformin	Metformin	Nigerian-German Chemicals Plc	Nigeria	B	04-6426	verified	FPD080521	HPLC	in specification	in specification	in specification	QMN 126
127	Diabetmin	Metformin	HOVID Bhd.	Malaysia	B	04-0810	verified	CB01603	HPLC	in specification	in specification	in specification	QMN 127
128	Biocipro	Ciprofloxacin	McCoy Pharma Pvt. Ltd.	India	B	B4-2452	not found	MP9420	HPLC	moderate deviation	moderate deviation	moderate deviation	QMN 128
129	Cenox	Ciprofloxacin	Mediios Laboratories Ltd.	India	B	04-3002	verified	M0063	HPLC	in specification	in specification	in specification	QMN 129

130	Nemel Cipro	Ciprofloxacin	NEMEL PHARMACEUTICALS LIMITED	Nigeria	B	B4-1405	verified	02E	HPLC	in specification	in specification	in specification	QMN 130
131	Cisepro-500	Ciprofloxacin	Fredun Pharmaceuticals Ltd.	India	B	B4-6330	not found	AA0037	HPLC	in specification	in specification	in specification	QMN 131
132	Flucozar	Fluconazole	J.DUNCAN HEALTHCARE PVT. LTD.	India	B	B4-2169	not found	J005B915	HPLC	in specification	not tested (capsule)	in specification	QMN 132
133	Johnbee Fluconazole Table	Fluconazole	Scott-Edil Pharmacia Ltd.	India	B	B4-6796	not found	XT9L037	HPLC	in specification	in specification	in specification	QMN 133
134	Berlin Fluconazole	Fluconazole	Kesar Pharma (P) Ltd.	India	B	C4-0748	not found	T20462	HPLC	in specification	in specification	in specification	QMN 134
135	AD-Fluconazole	Fluconazole	Globela Pharma Pvt. Ltd.	India	B	B4-1543	not found	20GT047	HPLC	in specification	in specification	in specification	QMN 135
136	Dexalab	Dexamethasone	Laborate Pharmaceuticals India Ltd.	India	B	04-5413	not found	KDSTE-003	HPLC	moderate deviation	moderate deviation	moderate deviation	QMN 136
137	Me cure Dexamethasone	Dexamethasone	Me Cure Industries Ltd.	Nigeria	B	A4-0201	verified	DC.227	HPLC	in specification	moderate deviation	moderate deviation	QMN 137
138	Nkoyo Dexamethasone	Dexamethasone	AR Lifesciences	India	B	04-8665	not found	L263	HPLC	extreme deviation	moderate deviation	extreme deviation	QMN 138
139	Pauco Dexamethasone tab	Dexamethasone	PAUCO Pharmaceutical Ind. Ltd.	Nigeria	B	A4-1036	not found	016	HPLC	moderate deviation	moderate deviation	moderate deviation	QMN 139
140	Loxaprim	Co-trimoxazole*	MAY&BAKER NIGERIA PLC	Nigeria	B	04-5567	verified	A170506	HPLC	in specification	in specification	in specification	QMN 140
141	Frusemide tablets shree ax	Furosemide	Mancare PHARMACEUTICALS PVT. LTD.	India	B	A4-0604	not found	TUK17	UV/Vis	in specification	in specification	in specification	QMN 141
142	Frusatex	Furosemide	Surelife Pharmaceutical Industries Ltd.	Nigeria	B	A11-0107	not found	028	UV/Vis	in specification	moderate deviation	moderate deviation	QMN 142
143	Nemegyl	Metronidazole	NEMEL PHARMACEUTICALS LIMITED	Nigeria	B	04-5326	not found	O1G	HPLC	in specification	in specification	in specification	QMN 143
144	Co-Trox	Co-trimoxazole*	VITABIOTICS (NIG.) LTD.	Nigeria	B	04-0061	not found	T59421	HPLC	in specification	in specification	in specification	QMN 144
145	Atenolol	Atenolol	ALPA LABORATORIES LTD.	India	B	B4-8238	verified	TE0183	HPLC	in specification	in specification	in specification	QMN 145
146	Metrozol	Metronidazole	VITABIOTICS (NIG.) LTD.	Nigeria	B	A4-6028	not found	T461119	HPLC	in specification	in specification	in specification	QMN 146
147	Cipxin-500	Ciprofloxacin	EUROLIFE HEALTHCARE PVT. LTD.	India	B	04-6293	not found	CPN046	HPLC	in specification	in specification	in specification	QMN 147 (1/2)
148	Camtaxone	Ceftriaxone	Shandong Xier Kangtai Pharm Co. Ltd	China	B	A4-7940	not found	20538	-	in specification	-	in specification	QMN 148
149	Pulmocef 500	Cefuroxime axetil	Micro Labs Ltd.	India	A	A4-1971	verified	PEFB0063	UV/Vis	in specification	in specification	in specification	QMN 149
150	Gecip	Ciprofloxacin	Jiangsu Ruinian Qianjin Pharmaceutical Co. Ltd.	China	A	B4-5856	not found	200417	HPLC	in specification	in specification	in specification	QMN 150
151	GG Dexamethasone Tablet	Dexamethasone	Jiangsu Pengyao Pharmaceutical Co. Ltd. (INC.)	China	A	A4-1598	not found	200821	HPLC	extreme deviation	moderate deviation	extreme deviation	QMN 151
152	Triaxin	Ceftriaxone	CSPC Zhongnuo Pharmaceuticals Co. Ltd.	China	A	04-8886	verified	659200615	-	in specification	-	in specification	QMN 152 (1/2)
153	Oxispa 500	Cefuroxime axetil	MEDICO REMEDIES LTD.	India	A	B4-6770	not found	OXS001	UV/Vis	in specification	in specification	in specification	QMN 153
154	Doncinat - 500	Cefuroxime axetil	BAROQUE PHARMACEUTICALS PVT. LTD.	India	A	A4-6907	verified	C010058	UV/Vis	in specification	in specification	in specification	QMN 154
155	Cipronol 500	Ciprofloxacin	Maxheal Laboratories Pvt. Ltd.	India	A	04-6340	not found	PL21007	HPLC	in specification	in specification	in specification	QMN 155
156	Ceftriaxone for injection	Ceftriaxone	Aqua Vitoe Laboratories	India	A	B4-3696	not found	B042020	-	in specification	-	in specification	QMN 156
157	Ciprofloxacin 500	Ciprofloxacin	Fidson Healthcare Plc.	Nigeria	A	A11-0403	not found	T2521001	HPLC	in specification	moderate deviation	moderate deviation	QMN 157
158	Xymatyl 500	Cefuroxime axetil	Adora Products Pvt. Ltd.	India	A	B4-5993	not found	HG303L20	UV/Vis	in specification	in specification	in specification	QMN 158
159	Nkoyo Fluconazole	Fluconazole	Mancare PHARMACEUTICALS PVT. LTD.	India	A	A4-0421	not found	TVB62	HPLC	in specification	in specification	in specification	QMN 159
160	Nkoyo Dexamethasone	Dexamethasone	McCoy Pharma Pvt. Ltd.	India	A	04-8665	not found	L20072	HPLC	moderate deviation	moderate deviation	moderate deviation	QMN 160
161	Ceftriaxone Injection	Ceftriaxone	Shanxi Zhongbao Shuguang Pharmaceutical Co., Ltd.	China	A	A4-9551	not found	210120	-	in specification	-	in specification	QMN 161
162	IVIM Ceftriaxone for Inject	Ceftriaxone	Scott-Edil Advance Research Laboratories & Education Ltd.	India	A	B4-4837	not found	1340Z081	-	in specification	-	in specification	QMN 162
163	Ciprobiotic-Forte	Ciprofloxacin	Emcure PHARMACEUTICALS LTD.	India	A	04-2307	not found	E16QP20046	HPLC	in specification	in specification	in specification	QMN 163
164	Diaformin	Metformin	BAL PHARMA LIMITED	India	A	B4-7950	not found	MTE42	HPLC	in specification	in specification	in specification	QMN 164
165	Glanil	Glibenclamide	Nigerian-German Chemicals Plc	Nigeria	A	04-2450	not found	FPD070321	HPLC	in specification	moderate deviation	moderate deviation	QMN 165
166	Gluformin	Metformin	Nigerian-German Chemicals Plc	Nigeria	A	04-6426	verified	FPC080121	HPLC	in specification	in specification	in specification	QMN 166
167	Clezide	Glibenclamide	Jiangsu Ruinian Qianjin Pharmaceutical Co. Ltd.	China	A	A4-2100	verified	190524	HPLC	moderate deviation	in specification	moderate deviation	QMN 167
168	Xasten	Dexamethasone	Jiangsu Pengyao Pharmaceutical Co. Ltd. (INC.)	China	A	04-6822	verified	200925	HPLC	moderate deviation	moderate deviation	moderate deviation	QMN 168
169	Cevmid	Ceftriaxone	Shandong Xier Kangtai Pharm Co. Ltd	China	A	A4-3304	not found	200954	-	in specification	-	in specification	QMN 169
170	Sopro Caplet	Ciprofloxacin	Jiangsu Pengyao Pharmaceutical Co. Ltd. (INC.)	China	A	B4-0053	not found	190819	HPLC	in specification	in specification	in specification	QMN 170
171	Vixa-Metformin	Metformin	Jiangsu Ruinian Qianjin Pharmaceutical Co. Ltd.	China	A	A4-2031	not found	191237	HPLC	in specification	in specification	in specification	QMN 171
172	Cefsafe-500	Cefuroxime axetil	missing	India	A	C4-0252	verified	HH108B21	UV/Vis	in specification	in specification	in specification	QMN 172
173	Eden Fluconazole	Fluconazole	IMPULSE PHARMA PVT LTD.	India	A	C4-0072	not found	200149	HPLC	in specification	not tested (capsule)	in specification	QMN 173

174	Famagyl	Metronidazole	PHAMATEX INDUSTRIES LIMITED	Nigeria	A	B4-2269	verified	T0157	HPLC	in specification	in specification	in specification	QMN 174
175	Pilotab	Ciprofloxacin	PHAMATEX INDUSTRIES LIMITED	Nigeria	A	B4-2270	verified	T1037	HPLC	in specification	in specification	in specification	QMN 175
176	Diapil	Metformin	PHAMATEX INDUSTRIES LIMITED	Nigeria	A	A11-0211	not found	T0082	HPLC	in specification	in specification	in specification	QMN 176
177	Flucox	Fluconazole	PHAMATEX INDUSTRIES LIMITED	Nigeria	A	B4-2972	verified	C1002	HPLC	in specification	not tested (capsule)	in specification	QMN 177
178	Licafur 500 Tablets	Cefuroxime axetil	BAROQUE PHARMACEUTICALS PVT. LTD.	India	A	B4-0098	verified	C010009	UV/Vis	in specification	in specification	in specification	QMN 178
179	Biophin Injection	Ceftriaxone	Intracin Pharmaceutical Pvt. Ltd.	India	A	04-6529	not found	20P10	-	in specification	-	in specification	QMN 179
180	Gluconorm SR 500	Metformin	LINCOLN PHARMACEUTICALS LTD.	India	A	A4-6949	verified	HB9003	HPLC	in specification	in specification	in specification	QMN 180
181	Galcipro 500	Ciprofloxacin	SKG-Pharma Limited	Nigeria	A	A4-0327	not found	2101	HPLC	moderate deviation	moderate deviation	moderate deviation	QMN 181
182	Primpex	Co-trimoxazole*	SKG-Pharma Limited	Nigeria	A	04-1959	not found	2136	HPLC	in specification	in specification	in specification	QMN 182
183	Metrotab 400	Metronidazole	SKG-Pharma Limited	Nigeria	A	04-9101	verified	0721	HPLC	in specification	in specification	in specification	QMN 183
184	Biophage 500	Metformin	SKG-Pharma Limited	Nigeria	A	A4-6597	verified	2120	HPLC	in specification	in specification	in specification	QMN 184
185	Avrotrim	Co-trimoxazole*	SKG-Pharma Limited	Nigeria	A	A4-4549	verified	2148	HPLC	in specification	in specification	in specification	QMN 185
186	Avrogyl	Metronidazole	SKG-Pharma Limited	Nigeria	A	A4-4725	verified	2107	HPLC	in specification	in specification	in specification	QMN 186
187	Avrocipro	Ciprofloxacin	SKG-Pharma Limited	Nigeria	A	A11-0786	verified	0221	HPLC	moderate deviation	moderate deviation	moderate deviation	QMN 187
188	Gauze Chloroquin	Chloroquine	Gauze Pharm. & Labs. Ltd.	Nigeria	A	A11-0076	not found	GCT031	HPLC; UV/Vis	in specification	in specification	in specification	QMN 188
189	Cyplox	Ciprofloxacin	MEDREICH LIMITED	India	A	04-3202	verified	B00045	HPLC	in specification	moderate deviation	moderate deviation	QMN 189
190	Glucophage 500 mg	Metformin	Merck S.L. Poligono Merck	Spain	A	04-6233	verified	E206246	HPLC	in specification	in specification	in specification	QMN 190
191	Zimatrim	Co-trimoxazole*	Gauze Pharm. & Labs. Ltd.	Nigeria	A	A11-0064	not found	ZGT037	HPLC	probably falsified	extreme deviation	probably falsified	QMN 191
192	Co-Trox	Co-trimoxazole*	VITABIOTICS (NIG.) LTD.	Nigeria	A	04-0061	not found	T17919	HPLC	in specification	in specification	in specification	QMN 192
193	Unigyl 200	Metronidazole	Unique Pharmaceuticals Ltd.	Nigeria	A	04-8426	verified	UGT9011	HPLC	in specification	in specification	in specification	QMN 193
194	Zimagil	Metronidazole	Gauze Pharm. & Labs. Ltd.	Nigeria	A	A11-0059	not found	ZGT091	HPLC	in specification	in specification	in specification	QMN 194
195	Zimagil	Metronidazole	Gauze Pharm. & Labs. Ltd.	Nigeria	A	A11-0059	not found	ZGT082	HPLC	in specification	in specification	in specification	QMN 195
196	Zimagil	Metronidazole	Gauze Pharm. & Labs. Ltd.	Nigeria	A	A11-0059	not found	ZGT089	HPLC	in specification	in specification	in specification	QMN 197
197	Metformin	Metformin	SWISS PHARMA PVT LTD.	India	A	C4-0429	not found	0211	HPLC	in specification	in specification	in specification	QMN 198
198	Tricophage - 500	Metformin	BAROQUE PHARMACEUTICALS PVT. LTD.	India	A	B4-2429	not found	G039009	HPLC	in specification	in specification	in specification	QMN 199
199	Axacef	Cefuroxime axetil	MEDREICH LIMITED	India	A	04-6027	not found	C00127	UV/Vis	in specification	in specification	in specification	QMN 200
200	Tribinat-500	Cefuroxime axetil	BAROQUE PHARMACEUTICALS PVT. LTD.	India	A	B4-2436	not found	C039009	UV/Vis	in specification	in specification	in specification	QMN 201
201	Zoxon	Ceftriaxone	MEDREICH LIMITED	India	A	04-9534	verified	C00073	-	in specification	-	in specification	QMN 202
202	Pocco Ceftriaxone Injectio	Ceftriaxone	Scott-Edil Advance Research Laboratories & Education Ltd.	India	A	B4-9368	verified	1340Z136	-	in specification	-	in specification	QMN 203
203	Zidek	Ceftriaxone	CSPC Zhongnuo Pharmaceuticals Co. Ltd.	China	A	B4-8006	not found	2005801	-	in specification	-	in specification	QMN 204
204	Nkoyo Dexamethasone	Dexamethasone	Mancare PHARMACEUTICALS PVT. LTD.	India	A	04-8665	not found	TVL55	HPLC	moderate deviation	moderate deviation	moderate deviation	QMN 205
205	Atenolol 50 mg Tablets	Atenolol	Scott-Edil Pharmacia Ltd.	India	A	B4-1651	verified	XT9L027	HPLC	in specification	in specification	in specification	QMN 206
206	Hydrex	Hydrochlorothiazide	JUHEL NIGERIA LIMITED	Nigeria	A	A4-1209	verified	0131	HPLC	in specification	in specification	in specification	QMN 207
207	New Divine Frusemide	Furosemide	New Divine Favour Pharmaceutical Industries Ltd.	Nigeria	A	A4-8776	not found	0029	UV/Vis	in specification	moderate deviation	moderate deviation	QMN 208
208	Fluconazole 150 mg	Fluconazole	Globela Pharma Pvt. Ltd.	India	A	B4-7568	not found	GT20110	HPLC	in specification	in specification	in specification	QMN 209
209	Trust Time Dexamethason	Dexamethasone	DUPEN LABORATORIES PVT. LTD.	India	B	B4-5651	verified	A009	HPLC	moderate deviation	moderate deviation	moderate deviation	QMN 210
210	Tamaflex 500	Ciprofloxacin	Maxtar Bio-Genics	India	A	A4-9436	not found	M3TGU1902	HPLC	in specification	in specification	in specification	QMN 211
211	Destrax	Dexamethasone	Jiangsu Pengyao Pharmaceutical Co. Ltd. (INC.)	China	A	B4-3876	not found	181201	HPLC	probably falsified	extreme deviation	probably falsified	QMN 212
212	Novalor caplets	Chloroquine	SKG-Pharma Limited	Nigeria	A	04-1442	not found	2001	HPLC; UV/Vis	in specification	extreme deviation	extreme deviation	QMN 213
213	Zoxon	Ceftriaxone	MEDREICH LIMITED	India	A	04-9534	verified	C00150	-	in specification	-	in specification	QMN 214
214	Zoxon	Ceftriaxone	MEDREICH LIMITED	India	A	04-9534	verified	C00069	-	in specification	-	in specification	QMN 215
215	Esidrex	Hydrochlorothiazide	DELPHARM L'AIGLE	France	B	missing	-	2OFA379	HPLC	in specification	in specification	in specification	QMN 216 (2/2)
216	Triaxin	Ceftriaxone	CSPC Zhongnuo Pharmaceuticals Co. Ltd.	China	A	04-8886	verified	659200616	-	in specification	-	in specification	QMN 217 (2/2)
217	Famagyl	Metronidazole	PHAMATEX INDUSTRIES LIMITED	Nigeria	A	B4-2269	verified	T1137	HPLC	in specification	in specification	in specification	QMN 218
218	Afrab Chloroquine	Chloroquine	Afrab-Chem Ltd.	Nigeria	A	A11-100076	verified	21252	HPLC; UV/Vis	in specification	in specification	in specification	QMN 219
219	Flucox	Fluconazole	PHAMATEX INDUSTRIES LIMITED	Nigeria	A	B4-2972	verified	C1005	HPLC	in specification	not tested (capsule)	in specification	QMN 220
220	Ciproheal Tablets	Ciprofloxacin	Maxheal Laboratories Pvt. Ltd.	India	A	04-7436	not found	CF21036	HPLC	in specification	in specification	in specification	QMN 221
221	Tionil	Glibenclamide	Merit Organics Ltd.	India	A	B4-7811	not found	T32004	HPLC	moderate deviation	in specification	moderate deviation	QMN 222

222	Nkoyosix	Furosemide	Mancare PHARMACEUTICALS PVT. LTD.	India	A	A4-9179	verified	TUL36	UV/Vis	in specification	in specification	in specification	QMN 223
223	Chloroquine	Chloroquine	JUHEL NIGERIA LIMITED	Nigeria	A	04-0171	not found	0006	HPLC; UV/Vis	in specification	in specification	in specification	QMN 224
224	Hydrex	Hydrochlorothiazide	JUHEL NIGERIA LIMITED	Nigeria	A	A4-1209	verified	0157	HPLC	in specification	in specification	in specification	QMN 225
225	Avrotrim	Co-trimoxazole*	SKG-Pharma Limited	Nigeria	A	A4-4549	verified	2205	HPLC	in specification	in specification	in specification	QMN 226
226	Adnil	Glibenclamide	Globela Pharma Pvt. Ltd.	India	A	B4-9967	not found	GT21094	HPLC	in specification	in specification	in specification	QMN 227
227	Gliben-J	Glibenclamide	JUHEL NIGERIA LIMITED	Nigeria	A	04-5735	not found	0041	HPLC	in specification	in specification	in specification	QMN 228
228	Tribinat-500	Cefuroxime axetil	BAROQUE PHARMACEUTICALS PVT. LTD.	India	A	B4-2436	not found	C031010	UV/Vis	in specification	in specification	in specification	QMN 229
229	Eden Fluconazole	Fluconazole	IMPULSE PHARMA PVT LTD.	India	A	C4-0072	not found	200149	HPLC	in specification	not tested (capsule)	in specification	QMN 230
230	AD-Fluconazole	Fluconazole	Globela Pharma Pvt. Ltd.	India	A	B4-1543	not found	20GT183	HPLC	in specification	in specification	in specification	QMN 231
231	AD-Fluconazole	Fluconazole	Globela Pharma Pvt. Ltd.	India	A	B4-1543	not found	20GT048	HPLC	in specification	in specification	in specification	QMN 232
232	Cefsafe-500	Cefuroxime axetil	missing	India	A	C4-0252	verified	HH1015B21	UV/Vis	in specification	in specification	in specification	QMN 233
233	Avrogyl	Metronidazole	SKG-Pharma Limited	Nigeria	A	A4-4725	verified	2201	HPLC	in specification	in specification	in specification	QMN 234
234	Ricogyl	Metronidazole	RICO PHARMACEUTICAL IND. LTD.	Nigeria	A	04-4590	not found	RGT002	HPLC	moderate deviation	in specification	moderate deviation	QMN 235
235	Ricotrin	Co-trimoxazole*	RICO PHARMACEUTICAL IND. LTD.	Nigeria	A	04-4589	not found	RTN001	HPLC	extreme deviation	extreme deviation	extreme deviation	QMN 236
236	Primpex	Co-trimoxazole*	SKG-Pharma Limited	Nigeria	A	04-1959	not found	2210	HPLC	in specification	in specification	in specification	QMN 237
237	Clezide	Glibenclamide	Jiangsu Ruinian Qianjin Pharmaceutical Co. Ltd.	China	A	A4-2100	verified	210331	HPLC	in specification	in specification	in specification	QMN 238
238	Metrotab 200	Metronidazole	SKG-Pharma Limited	Nigeria	A	04-9936	verified	2202	HPLC	in specification	in specification	in specification	QMN 239
239	Glanil	Glibenclamide	Nigerian-German Chemicals Plc	Nigeria	A	04-2450	not found	FPJ070121	HPLC	in specification	extreme deviation	extreme deviation	QMN 240
240	Clamide	Glibenclamide	HOVID Bhd.	Malaysia	A	04-4015	verified	CA06595	HPLC	in specification	in specification	in specification	QMN 241
241	Glanil	Glibenclamide	Nigerian-German Chemicals Plc	Nigeria	A	04-2450	not found	FPD070421	HPLC	in specification	moderate deviation	moderate deviation	QMN 242
242	Hydrex	Hydrochlorothiazide	JUHEL NIGERIA LIMITED	Nigeria	A	A4-1209	verified	0158	HPLC	in specification	in specification	in specification	QMN 243
243	Unigyl 200	Metronidazole	Unique Pharmaceuticals Ltd.	Nigeria	A	04-8426	verified	UGT1067	HPLC	in specification	in specification	in specification	QMN 244
244	Gliben-J	Glibenclamide	JUHEL NIGERIA LIMITED	Nigeria	A	04-5735	not found	0043	HPLC	in specification	in specification	in specification	QMN 245
245	Sivonat	Cefuroxime axetil	Saga Lifesciences Limited	India	A	C4-0482	not found	SAKUO22101	UV/Vis	in specification	moderate deviation	moderate deviation	QMN 246
246	Eden Atenolol	Atenolol	IMPULSE PHARMA PVT LTD.	India	A	B4-6760	not found	210917	HPLC	in specification	extreme deviation	extreme deviation	QMN 247
247	Triflucon	Fluconazole	BAROQUE PHARMACEUTICALS PVT. LTD.	India	A	A4-6953	verified	G031002	HPLC	in specification	not tested (capsule)	in specification	QMN 248
248	Ratenol	Atenolol	OSAKA Pharmaceuticals Pvt. Ltd.	India	A	A4-3899	not found	OS033	HPLC	extreme deviation	moderate deviation	extreme deviation	QMN 249
249	Eden Atenolol	Atenolol	IMPULSE PHARMA PVT LTD.	India	A	B4-6760	not found	210916	HPLC	in specification	extreme deviation	extreme deviation	QMN 250
250	M&B Chloroquine	Chloroquine	MAY&BAKER NIGERIA PLC	Nigeria	A	04-0705	not found	A200600	HPLC; UV/Vis	in specification	in specification	in specification	QMN 251
251	Hydrex	Hydrochlorothiazide	JUHEL NIGERIA LIMITED	Nigeria	A	A4-1209	verified	0127	HPLC	moderate deviation	in specification	moderate deviation	QMN 252
252	Unigyl 200	Metronidazole	Unique Pharmaceuticals Ltd.	Nigeria	A	04-8426	verified	UGT1103	HPLC	in specification	in specification	in specification	QMN 253
253	GG Dexamethasone Tablet	Dexamethasone	Jiangsu Pengyao Pharmaceutical Co. Ltd. (INC.)	China	A	A4-1598	not found	200821	HPLC	extreme deviation	moderate deviation	extreme deviation	QMN 254
254	Hydrex	Hydrochlorothiazide	JUHEL NIGERIA LIMITED	Nigeria	A	A4-1209	verified	0157	HPLC	in specification	in specification	in specification	QMN 255
255	Chloroquine Tablets	Chloroquine	emzor Pharmaceutical Industries Ltd.	Nigeria	A	04-1218	not found	1819Z	HPLC; UV/Vis	in specification	in specification	in specification	QMN 256
256	Loxagyl 200	Metronidazole	MAY&BAKER NIGERIA PLC	Nigeria	A	04-0283	not found	A201841	HPLC	in specification	in specification	in specification	QMN 257
257	Nkoyo Dexamethasone	Dexamethasone	Astamed Healthcare (I) Pvt. Ltd.	India	A	04-8665	not found	1008	HPLC	moderate deviation	extreme deviation	extreme deviation	QMN 258
258	Nkoyo Dexamethasone	Dexamethasone	Maxwell Life Science Pvt. Ltd.	India	A	04-8665	not found	LT110	HPLC	moderate deviation	moderate deviation	moderate deviation	QMN 259
259	Nkoyo Fluconazole	Fluconazole	Maxheal Laboratories Pvt. Ltd.	India	A	A4-0421	not found	KF21001	HPLC	in specification	moderate deviation	moderate deviation	QMN 260
260	Cipxin-500	Ciprofloxacin	EUROLIFE HEALTHCARE PVT. LTD.	India	B	04-6293	not found	CPN048	HPLC	in specification	not tested (not enough tabl	in specification	QMN 261 (2/2)

Supplementary Table S3. Results of the examined PIN codes for the Mobile Authentication Service Scheme.

**Product name**	**NAFDAC Reg. No**	**Tested MAS PIN code***	**Short phone number or website for MAS provider[#]**	**SMS response text received**	**Response complete[$]**	**Response correct**	**Comments**
Biophage 500	A4-6597	3037252567259	Sproxil (www.sproxil.com/verify)	(no response)			
Oxispa 500	A4-4096	2003971268069	Sproxil (www.sproxil.com/verify)	(no response)			
**Tribinat-500** (cefuroxime axetil tablets)	B4-2436	441101032981	www.1393.co (M-Pedigree)	**GENUINE Triclav Syrup, 70ml bottle, Amoxicillin 200mg & Clavulanate 28.5mg BATCH: Po39008, EXP: Dec/2021 Quality guaranteed**	No	**No**	**Incorrect product identified**
**Tricophage - 500** (metformin tablets)	B4-2429	101152044312	www.1393.co (M-Pedigree)	**GENUINE Triclav Syrup, 70ml bottle, Amoxicillin 200mg & Clavulanate 28.5mg BATCH: Po39008, EXP: Dec/2021 Quality guaranteed**	No	**No**	**Incorrect product identified** Expired 12/2022, but no warning
**Triflucon** (fluconazole tablets)	A4-6953	133551738573	www.1393.co (M-Pedigree)	**GENUINE Triclav Syrup, 70ml bottle, Amoxicillin 200mg & Clavulanate 28.5mg BATCH: Po39008, EXP: Dec/2021 Quality guaranteed**	No	**No**	**Incorrect product identified**
Glulife-500 mg	A4-6354	304924177735	www.1393.co (M-Pedigree)	This is an Original Product Marketed by Company: **Maydon Pharmaceuticals LTD**. For additional info, call 08039012030 or email nig@mpedigree.net.	No	**No**	**Incorrect MAH stated** Expired 03/2023, but no warning
Cenox	04-3002	2003585274982	Sproxil (38353)	Genuine PRODUCT Your PIN:2003585274982 Problem? Call 08039012929	No	+/-	Expired 05/2023, but no warning

Product name	NAFDAC Reg. No	Tested MAS PIN code*	Short phone number or website for MAS provider#	SMS response text received	Response complete$	Response correct	Comments
Ciprobiotic-Forte	04-2307	2003540121858	Sproxil (38353)	Genuine PRODUCT<br>Your PIN:2003540121858<br>Problem? Call 08039012929	No	+/-	Expired 02/2023, but no warning
Ciproheal Tablets	04-7436	2003 38784 7342	Sproxil (38353)	Genuine PRODUCT<br>Your PIN:2003387847342<br>Problem? Call 08039012929	No	+/-	
Doncinat - 500	A4-6907	2003557512765	Sproxil (38353)	Genuine PRODUCT<br>Your PIN:2003557512765<br>Problem? Call 08039012929	No	+/-	
Elcexone	B4-7427	2003956193498	Sproxil (38353)	Genuine PRODUCT<br>Your PIN:2003956193498<br>Problem? Call 08039012929	No	+/-	
Oxispa 500	B4-6770	2003658753644	Sproxil (38353)	Genuine PRODUCT<br>Your PIN:2003658753644<br>Problem? Call 08039012929	No	+/-	
Oxispa 500	B4-6770	2003730651937	Sproxil (38353)	Genuine PRODUCT<br>Your PIN:2003730651937<br>Problem? Call 08039012929	No	+/-	
Pocco Ceftriaxone Injection	B4-9368	2003 01148 8354	Sproxil (38353)	Genuine PRODUCT<br>Your PIN:2003011488354<br>Problem? Call 08039012929	No	+/-	
Pocco Ceftriaxone Injection	B4-9368	2003 57420 1148	Sproxil (38353)	Genuine PRODUCT<br>Your PIN:2003574201148<br>Problem? Call 08039012929	No	+/-	
Biophage 500	A4-6597	3037 22711 7565	Sproxil (38353)	Genuine Biophage product<br>Your PIN:3037227117565<br>Problem? Call 08039012929	No	+/-	
Pilotab	B4-2270	3037 29691 4132	Sproxil (38353)	Genuine Pharmatex Product<br>Your PIN: 3037296914132<br>Problem? Call 08039012929	No	+/-	
Sopro Caplet	B4-0053	3036 8990 66358	Sproxil (38353)	Genuine Sopro Tablet 500mg<br>PIN:3036899066358<br>NRN:B4-0053<br>Problem? Call 08039012929	No	Yes	Expired 08/2022, but no warning

Product name	NAFDAC Reg. No	Tested MAS PIN code*	Short phone number or website for MAS provider#	SMS response text received	Response complete$	Response correct	Comments
Tamaflex 500	A4-9436	2003 17177 3226	Sproxil (38353)	Genuine Tamar & Pharez Products<br>Your PIN:2003171773226<br>Problem? Call 08039012929	No	Yes	Expired 06/2022, but no warning
Ceftriaxone for injection	B4-3696	118784041873	www.1393.co (M-Pedigree)	GENUINE Product, 1 Pack, Approved batch	No	+/-	Expired 05/2022, but no warning
Harvad Cefuroxime tablets	B4-7976	297807456353	www.1393.co (M-Pedigree)	GENUINE Product, 1 Pack, Genuine Batch	No	+/-	
Harvad Cefuroxime tablets	B4-7976	838903420108	www.1393.co (M-Pedigree)	GENUINE Product, 1 Pack, Genuine Batch	No	+/-	
Harvad Cefuroxime tablets	B4-7976	157942465615	www.1393.co (M-Pedigree)	GENUINE Product, 1 Pack, Genuine Batch	No	+/-	
Roxirite	C4-1189	339243381288	www.1393.co (M-Pedigree)	GENUINE Product, 1 Pack, Genuine Batch	No	+/-	
Roxirite	C4-1189	808405138219	www.1393.co (M-Pedigree)	GENUINE Product, 1 Pack, Genuine Batch	No	+/-	
Xymatyl 500	B4-5993	258811498601	www.1393.co (M-Pedigree)	GENUINE Product, 1 Pack, Genuine Batch	No	+/-	
Xymatyl 500	B4-5993	170309522090	www.1393.co (M-Pedigree)	GENUINE Product, 1 Pack, Genuine Batch	No	+/-	
Flucoxiom-150	C4-1024	302477155688	www.1393.co (M-Pedigree)	GENUINE Product, Single pack, Genuine Batch	No	+/-	
Pulmocef 500	A4-1971	111878197880	www.1393.co (M-Pedigree)	GENUINE Product, Genuine batch, NAFDAC approved	No	+/-	Expired 05/2023, but no warning

Product name	NAFDAC Reg. No	Tested MAS PIN code*	Short phone number or website for MAS provider#	SMS response text received	Response complete$	Response correct	Comments
Ugolife Ciprofloxacin-500	B4-7467	891830284925	www.1393.co (M-Pedigree)	This is an Original Product Marketed by Company: Kesar Pharma Pvt Ltd. For additional info, call 08039012030 or email nig@mpedigree.net.	No	+/-	
Sivonat	C4-0482	293978918492	www.1393.co (M-Pedigree)	This is an Original Product Marketed by Company: Saga. For additional info, call 08039012030 or email nig@mpedigree.net.	No	+/-	Expired 01/2023, but no warning
Axacef	04-6027	118955608358	www.1393.co (M-Pedigree)	This is an Original Product Marketed by Company: Sanofi Aventis. For additional info, call 08039012030 or email nig@mpedigree.net.	No	+/-	Expired 05/2023, but no warning
Cyplox	04-3202	202727241800	www.1393.co (M-Pedigree)	This is an Original Product Marketed by Company: Sanofi Aventis. For additional info, call 08039012030 or email nig@mpedigree.net.	No	+/-	Expired 12/2022, but no warning
Zoxon	04-9534	537656158458	www.1393.co (M-Pedigree)	This is an Original Product Marketed by Company: Sanofi Aventis. For additional info, call 08039012030 or email nig@mpedigree.net.	No	+/-	Expired 04/2023, but no warning
Zoxon	04-9534	798019221133	www.1393.co (M-Pedigree)	This is an Original Product Marketed by Company: Sanofi Aventis. For additional info, call 08039012030 or email nig@mpedigree.net.	No	+/-	
Zoxon	04-9534	307043651856	www.1393.co (M-Pedigree)	This is an Original Product Marketed by Company: Sanofi Aventis. For additional info, call 08039012030 or email nig@mpedigree.net.	No	+/-	Expired 04/2023, but no warning
Tricophage - 500	B4-2429	605171935945	www.1393.co (M-Pedigree)	This is an Original Product Marketed by Company: Tricare Pharmaceutical Nig, Ltd. For additional info, call 08039012030 or email nig@mpedigree.net.	No	+/-	Expired 12/2022, but no warning
Licafur 500 Tablets	B4-0098	130265549005	www.1393.co (M-Pedigree)	This is an Original Product Marketed by Company: Zolon Healthcare Limited. For additional info, call 08039012030 or email nig@mpedigree.net.	No	+/-	Expired 05/2022, but no warning

Product name	NAFDAC Reg. No	Tested MAS PIN code*	Short phone number or website for MAS provider#	SMS response text received	Response complete$	Response correct	Comments
Tribinat-500	B4-2436	444025028540	www.1393.co (M-Pedigree)	GENUINE NAFDAC Approved Pharmaceutical Product, Marketed and Distributed by, Tricare Pharmaceutical Nigeria Ltd	No	+/-	Expired 01/2023, but no warning
Cisepro-500	B4-6330	246882900773	www.1393.co (M-Pedigree)	GENUINE Cisepro 500 , 1X10, Ciprofloxacin Tablets USP 500 mg<br>BATCH: AA0036 - AA0038, EXP: Dec/2023	No	Yes	
Cisepro-500	B4-6330	278007167152	www.1393.co (M-Pedigree)	GENUINE Cisepro 500 , 1X10, Ciprofloxacin Tablets USP 500 mg<br>BATCH: AA0036 - AA0038, EXP: Dec/2023	No	Yes	
Ibu Ciprofloxacin - 500	C4-0487	189229109167	www.1393.co (M-Pedigree)	GENUINE IBU CIPROFLOXACIN 500 TABLETS, 1x10, CIPROFLOXACIN HYDROCHLORIDE USP 500 mg<br>BATCH: T21001 - T21008, EXP: Dec/2023	No	Yes	
Nemel Cipro	B4-1405	289128501270	www.1393.co (M-Pedigree)	GENUINE Nemel Cipro, 1x10 caplets, Ciprofloxacin 500mg<br>BATCH: 02E, EXP: Apr/2026	No	Yes	
Gecip	B4-5856	5262 8948 118465	20966 (UBQ-t/Kezzler)	Original Gecip from Geneith Pharm Problem? Call 09095966343	No	Yes	Expired 04/2023, but no warning
Ciprofloxacin	B4-1176	ZYA994KE	38351 (Pharmasecure)	Warning! this product CIPROFLOXACIN TABLETS USP 500 MG expired on Feb-2023. Please return product to Chemist.	No	Yes	

Abbreviations: NAFDAC, National Agency for Food and Drug Administration and Control. MAS, Mobile Authentication Service.

* For 15 samples, several packages were available, each carrying a different MAS PIN code. A maximum of three MAS PINs were tested for one sample; in this table, only the first of the three tested MAS PINs is listed. For all these 15 samples, the responses received for the second and the third sample were identical to that received for the first sample.

# In addition to the 46 listed samples, one sample carried a PIN code by www.chekkit.app. This Service Provider is not listed by NAFDAC and was not tested.

$ According to the NAFDAC Guidelines for the Mobile Authentication Service (MAS) Scheme of 2018, the following minimum information must be contained in the SMS response:

- classification as "genuine product" or "Product not verifiable"
- product name
- NAFDAC registration number
- expiry date
- batch number
- helpline for further information)
